# Dietary consumption trajectory profiles over time of French adults from the NutriNet-Santé cohort (2014–2022): multicriteria analysis of sustainability

**DOI:** 10.1186/s12966-025-01777-w

**Published:** 2025-06-13

**Authors:** Hafsa Toujgani, Juhui Wang, Elie Perraud, Julia Baudry, Justine Berlivet, Benjamin Allès, Hélène Fouillet, Serge Hercberg, Mathilde Touvier, Denis Lairon, Philippe Pointereau, Christian Couturier, François Mariotti, Emmanuelle Kesse-Guyot, the TRANSFood Consortium, Aurélien Chayre, Helene Charreire, Jean-Francois Huneau, Laurent Muller, Sabrina Teyssier, Sylvaine Berger, Thierry Feuillet

**Affiliations:** 1 Université Sorbonne Paris Nord and Université Paris Cité, Inserm, INRAE, CNAM, Center of Research in Epidemiology and StatisticS (CRESS), Nutritional Epidemiology Research Team (EREN), 93017 Bobigny, France; 2 Université Paris-Saclay, AgroParisTech, INRAE, UMR PNCA, 91120 Palaiseau, France; 3 Aix Marseille Université, Inserm, INRAE, C2VN, 13005 Marseille, France; 4 Solagro, 75, Voie TOEC, CS 27608, F-31076, Cedex 3 Toulouse, France

**Keywords:** Consumption trajectories, Greenhouse gas emissions, Nutritional quality, Socioeconomic factors, Diet-related diseases

## Abstract

**Background:**

Dietary patterns significantly impact climate change and morbidity, making transitions to sustainable diets urgent. Few studies explore repeated dietary measures collected over several years or variations across distinct consumer profiles characterized by sociodemographic and dietary patterns.

**Objective:**

Our study aims to identify dietary trajectory profiles in French adults (2014–2022), assessing environmental, nutritional, and health outcomes.

**Methods:**

Consumption data from 17,187 NutriNet-Santé cohort participants (52% women, average age 48y, SD = 16) were collected via food frequency questionnaires in 2014 (weighted to the French Census), 2018, and 2022. Dietary trajectory profiles were modeled using Group-based multi-trajectory modeling based on principal component analysis of energy-adjusted consumption data. Associations with environmental (greenhouse gas (GHG) emissions) and nutritional (adherence to French dietary guidelines, diet quality index) dimensions were assessed via multivariable mixed models. Health impacts were evaluated as Disability-Adjusted Life Years (DALYs)avoided, using the Comparative Risk Assessment approach.

**Results:**

Six dietary trajectory profiles (P) with distinct starting diets and evolutions were identified. P0 had average intakes, while P1 and P5 were meat-focused, with P5 showing the highest animal consumption. P3 and P4 leaned plant-based, with P4 maintaining high fish and plant intakes, and P3 increasing ruminant meat. P2 initially high in salty or sweet fatty foods, shifted significantly toward plant-based diets. Over time, GHG emissions decreased (− 5% to − 14%), diet quality (PNNS-GS2 score) improved (+ 12% to + 174%), and health risks declined in four profiles due to reduced red meat and higher whole grains/fruits but increased for P4 and P5 due to processed meat.

**Conclusion:**

These profiles reflect diverse population segments with distinct dietary profiles and degrees of sustainability improvements. However, significant advancements remain limited, highlighting the need for further research on economic, psychological, and cultural factors to guide sustainable changes.

**Trial Registration:**

Trial registration number: NCT03335644. URL of registration: https://clinicaltrials.gov/study/NCT03335644?id=NCT03335644&rank=1

**Supplementary Information:**

The online version contains supplementary material available at 10.1186/s12966-025-01777-w.

## Highlights

• Six distinct profiles of 8y-dietary trajectories were identified, characterized by specific starting diets and subsequent evolution.

• With varying magnitude, GHG emissions decreased (−5% to −14%), and diet quality improved (+12% to +174% in PNNS-GS2 score) for all profiles.

• Health risks declined in four profiles due to reduced red meat and higher whole grains/fruits but increased in two profiles due to higher processed meat intake.

**Supplementary Information:**

The online version contains supplementary material available at 10.1186/s12966-025-01777-w.

## Background

Dietary consumption patterns are a significant driver of climate change, as food systems account for nearly one-third of global greenhouse gas (GHG) emissions [1]. Despite their crucial role, dietary consumption often remains underrepresented in climate policies that prioritize production-focused interventions [2]. This oversight is alarming given the transgression of six planetary boundaries, including climate change, biosphere integrity, and biogeochemical flows, which now operate beyond safe ecological limits [3–6]. Among these, climate change and biosphere integrity are approaching irreversible thresholds due to self-reinforcing feedbacks, such as CO₂ persistence and species extinction rates exceeding 100 extinctions per million species-years [6]. Urgent shifts in food consumption are critical to mitigate these systemic risks, as dietary changes directly impact land use, nitrogen cycles, and carbon emissions [7, 8]. According to the Intergovernmental Panel on Climate Change [9], aligning mitigation strategies with local consumption behaviors and cultural contexts is key to enhancing effectiveness [2] and achieving the Sustainable Development Goals by 2030 [10].

In addition to their environmental impact, diets also play a pivotal role in the global burden of morbidity. The Global Burden of Disease study [11] reveals that suboptimal diets cause more deaths than any other risk factor, including tobacco smoking, and that one in five deaths around the world could be prevented by improving dietary habits. Non-optimal intake of whole grains, fruits, and excessive sodium accounts for over 50% of diet-related deaths and 66% of disability-adjusted life years (DALYs).

Thus, although the transition to sustainable diets [12] is urgently needed, reconciling the four key dimensions (environment, nutrition and health, economy, and culture) presents a complex challenge. This difficulty is exacerbated by disciplinary fragmentation, which limits the development of integrated solutions that are both effective and acceptable to consumers [13]. While evidence suggests that dietary changes aimed at reducing non-communicable diseases can also support the achievement of international environmental goals [14, 15], a thorough understanding of current dietary trends is essential to identify existing gaps, monitor progress, and inform targeted strategies for promoting healthier and more sustainable diets.

While national food surveys provide valuable insights, they are often limited by the lack of longitudinal data, limiting the ability to analyze individual characteristics with temporal dietary changes. Furthermore, they frequently fail to assess diets from a sustainability perspective and primarily focus on population-level trends, overlooking variations within specific consumer segments. This knowledge gap is particularly evident in longitudinal data, which remains scarce and outdated [16–18], which limits the ability to focus on recent trends within the context of the ecological crisis.

Based on individual longitudinal data from the French population-based cohort NutriNet-Santé, our previous work explored the associations between food group consumption trends and socioeconomic factors in the general population [19]. Based on these findings, we hypothesized that dietary changes likely vary across different population segments, with distinct trajectories emerging. Understanding these trajectories is crucial for providing more precise documentation that could inform the development of targeted actions tailored to diverse consumer profiles'specific identities and needs.

In this context, our study aims to analyze recent trends in food consumption changes in the NutriNet-santé cohort by identifying various food trajectory profiles over 8 years (2014–2022). This segmentation approach allows for a detailed examination of the evolving dietary patterns and their impacts on sustainability. We further assess these profiles through sustainability indicators, focusing on environmental, nutritional, and health outcomes.

## Methods and data

### Study population

This research employed longitudinal observational data from 2014 to 2022, derived from a subset of the NutriNet-Santé cohort study participants. Launched in May 2009, the NutriNet-Santé study is an online cohort study designed to explore the determinants of diet, nutritional status, physical activity, and their relationships with health outcomes [20]. The study includes adult participants residing in France who have internet access and were recruited voluntarily. Participants must complete annual or biannual questionnaires addressing socioeconomic status, lifestyle, anthropometry, diet, and physical activity habits [21]. Additional non-mandatory questionnaires are administered periodically. Gender, occupational status, income, place of residence, physical activity levels, and smoking habits are all self-reported through validated questionnaires [21]. The NutriNet-Santé study complies with the principles outlined in the Helsinki Declaration and has received validation from both the Inserm Ethical Evaluation Committee (CEEI) (no. 0000388FWA00005831) and the National Committee for Information Technology and Freedom (CNIL) (nos. 908450 and 909,216). The study is also registered on ClinicalTrials.gov (NCT03335644).

This study was based on an initial sample of n = 29,195 participants residing within mainland France who completed the Org-FFQ in 2014, thus enabling the computation of a weighting procedure described below. Those considered under- or over-reporters for energy intake were excluded as previously published [22]. A subset of n = 17,187 participants who also completed the Org-FFQ in 2018 and/or 2022 was selected from the initial sample and used in constructing the typology described below. A flowchart is provided in Supplemental Fig. 1.

### Assessment of food group consumption

**Fig. 1:**
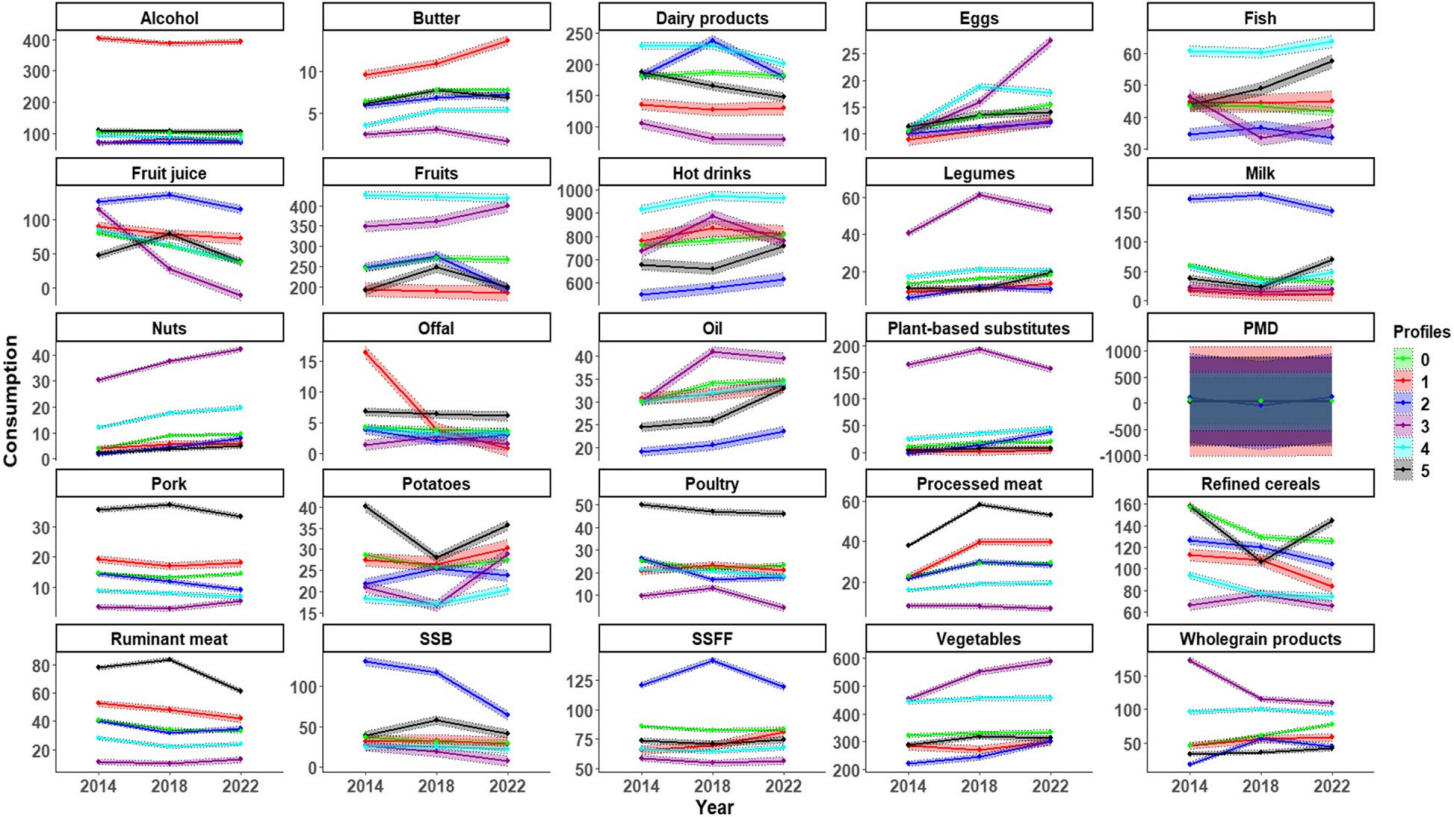
Trajectories of change in food group consumption (g/d) (2014 to 2022) by profile, *n* = 17,187, NutriNet-Santé Study^*1,2,3*^. Food groups are composed as follows: Ruminant meat include beef and lamb; Dairy products include yogurts, fresh cheese and cheese; Animal substitutes include tofu, soy-based meat substitute and vegetable patties, soy-based yogurt, soy-based milk; Vegetables include all vegetables and soups; Fruit include fresh fruit, fruit in syrup and compote, dried fruit and seeds; Fish include fatty and lean fish, mollusks, and crustaceans; Potatoes include other tubers; Refined cereals include breakfast cereal low in sugar, bread, semolina, rice and pasta; SSFF (salty or sweet and fatty foods) include croissants, pastries, chocolate, biscuits, milky dessert, ice cream, honey and marmalade, cakes, chips, salted oilseeds, salted biscuits; PMD (prepared and mixed dishes) include sandwich, prepared foods such as pizza, hamburger, ravioli, panini, salted pancake, etc.; Oil include plant-based oils and ready-to-use salad dressing, mayonnaise or cream-based sauces, sour cream and all fat-based sauces; Hot drinks include tea, infusions, milk consumed with tea/coffee; SSB (sugar-sweetened beverages) include fruit nectar, syrup, soda (with or without sugar), plant-based beverages. 1 Abbrevations: SSFF, Sweetened/Salty and Fatty Foods. SSB, Sugar-Sweetened Beverages. PMD, Prepared and Mixed Dishes. ^2^For each of the 25 food groups, the values shown are the least squares means (with 95% confidence intervals) of consumption in grams per day, adjusted for sex, age, and total energy intake, as estimated from the mixed-effects models. The y-axis represents these adjusted mean intakes for each trajectory profile and time point (2014, 2018, 2022).^3^The x-axis represents time (2014; 2018; 2022).

Food consumption data were collected in 2014, 2018, and 2022 using an Organic Food Frequency Questionnaire (Org-FFQ), which includes 264 organic and conventional food items. Specifically, for each of the 264 items in the questionnaire, participants were asked to indicate their frequency of consumption and the quantity consumed of organic food items over the past year, using a 5-point ordinal scale. Further details on this method are provided in a previous study [22] and are summarized in Supplemental Method 1. For the current study, a classification into 25 food groups has been established based on their nutritional value and contents and the greenhouse gas emissions related to their production, as follows: ruminant meat (including beef and lamb), pork, offal, processed meat, poultry, fish, eggs, dairy products (excluding milk), milk, plant-based substitutes, vegetables, fruits, fruit juices, legumes, wholegrain products, nuts, potatoes, refined cereals, prepared and mixed dishes (PMD, including sandwich, prepared foods such as pizza, hamburger, ravioli, panini, salted pancake, etc.), salty or sweet and fatty foods (SSFF), sugar-sweetened beverages (SSBs), hot drinks, alcoholic beverages, butter, and oils. Detailed information on the composition of the food groups is provided in the legend of Fig. 1. Total daily energy intake (TEI) and nutrient intakes were calculated using the food composition table designed for the NutriNet-Santé study [23].

### Environmental data

Greenhouse gas (GHG) emissions (kg CO2eq) were assessed using the DIALECTE tool [24]. Developed by Solagro, DIALECTE tool is designed to evaluate the environmental performance of French farms comprehensively. The Life Cycle Assessment method was applied to 60 raw agricultural products, completed by literature data for 32 products. DIALECTE focuses exclusively on the agricultural production stage and differentiates between organic and conventional products [25]. More details are provided in Supplemental Material 2. GHG emissions were selected as the primary indicator for assessing environmental impact due to their well-established relevance to climate change and their widespread use in the literature as a core metric for evaluating the environmental footprint of food systems [26–28]. Food production is responsible for approximately one-quarter to one-third of global GHG emissions, making it a major driver of planetary boundary transgression related to climate change [1, 28]. Furthermore, GHG emissions data are widely available and allow for robust cross-study and cross-country comparisons [26, 27]. Given these factors, GHG emissions were considered a representative and policy-relevant indicator for this analysis.

### Dietary quality scores

Two dietary indexes were computed. Adherence to the French dietary guidelines was assessed using the PNNS-GS2 score [29], which evaluates the intake of 13 food groups categorized as either healthy (to be encouraged) or unhealthy (to be limited), based on the 2017 recommendations of the High Council of Public Health [30]. Points are awarded for meeting recommended intakes of healthy foods and deducted for excessive consumption of foods to be limited, with additional weighting reflecting the strength of evidence linking each component to health outcomes. The score also incorporates a penalty for excessive energy intake and considers organic food consumption for certain plant-based groups. The final score provides a comprehensive measure of overall dietary quality, with details of the scoring system and reference portions provided in Supplemental Material 3.

Diet quality was assessed using the Comprehensive Diet Quality Index (cDQI), which combines two sub-scores: the Plant-based Diet Quality Index (pDQI) and the Animal-based Diet Quality Index (aDQI) [31]. The pDQI evaluates the consumption of eleven plant-based food groups, while the aDQI covers six animal-based food groups. Each food component sub-score ranges from 0 to 5. For each group, foods are classified as either healthy or unhealthy based on current scientific evidence and international dietary guidelines [31]. Scores are assigned proportionally to intake: higher consumption of healthy foods and lower consumption of unhealthy foods yield higher scores. The final cDQI score is the sum of the pDQI and aDQI, ranging from 0 to 85, with higher values reflecting better overall diet quality. Details of the food groups, scoring system, and reference intakes are provided in Supplemental Material 3.

### Statistical analysis

#### Modeling the typology of food transition trajectories

The construction of dietary consumption trajectory profiles was conducted in three main steps: The first step involved creating a longitudinal database synthesizing individual consumption information across 25 food groups. Initially, a Principal Component Analysis (PCA) was performed on the 25 food groups using the baseline sample in 2014 (n = 29,195). Those considered as under- or over-reporters for energy intake were excluded as previously published [22]. This sample was chosen to capture the broad diversity of dietary patterns within the population. The PCA identified new axes (principal components) that summarize the main dietary patterns in the population.

The 2014 dietary data were adjusted for total energy intake (TEI) using the residual method [32] and weighted using the iterative proportional fitting procedure according to 2009 national census data [33]. These weights were computed using data on age, occupational status, educational level, area of residence, presence of children (< 18 years), and marital status. This weighting method aimed to mitigate the low generalizability associated with the volunteer sample of the NutriNet-Santé cohort and to enhance its representativeness of the French population. A weighting factor was calculated for each individual, reflecting their probability of inclusion in a representative sample [33].

In the second step, the eigenvector matrix (component loadings) derived from the 2014 PCA was used to project dietary data from 2018 and 2022 onto the same principal component space, to ensure comparability of dietary pattern scores across all years. Specifically, for each participant and time point, energy-adjusted food group intakes were multiplied by the 2014 PCA loadings to calculate individual component scores. For this step, a subset of n = 17,187 participants was selected from the initial sample (n = 29,195), including individuals who completed the Org-FFQ in 2018 and/or 2022.

The third step involved modeling dietary consumption trajectory profiles. Based on the PCA scores for each individual at each time point (2014, 2018, and/or 2022), the Group-Based Multi-Trajectory Modeling (GBMT) method was employed to identify trajectory profiles. A first-degree model adjusted for age, sex, and TEI was used. Further methodological details related to the trajectories’ analysis are available in Supplemental Material 4.

#### Description and comparison of trajectory profiles

The identified profiles were described according to the socio-demographic characteristics reporting mean (SD) for continuous variables or % for categorical variables. Means comparison across clusters was performed using Pearson's Chi-square test for categorical variables and ANOVA test for continuous variables.

A multicriteria analysis of profiles’ sustainability was conducted, addressing two of the four dimensions of sustainable diets as defined by the FAO [12]: environmental impact and health/nutrition. The environmental dimension was assessed using GHG emissions, while the nutritional dimension was evaluated through compliance with French dietary guidelines (PNNS-GS2) and diet quality indices (cDQI, aDQI, pDQI). Additional indicators included the proportion of plant-based proteins and organic food in the diet, both of which are associated with documented health and environmental benefits. Higher plant-based protein intake is linked to a reduced risk of chronic diseases and lower greenhouse gas emissions compared to animal-based proteins [8, 15, 34–37]. Similarly, greater consumption of organic foods is associated with reduced exposure to synthetic pesticides and environmental benefits such as lower chemical pollution and enhanced biodiversity [38–43].

Analyses of food consumption changes in the 25 food groups and changes in indicators related to sustainability were conducted using multivariable mixed-effects models. For each model, the dependent variable was the consumption of a given food group or indicator, while the independent variables included trajectory profile, time, their interaction, as well as sex, age, and total energy intake (TEI). Random effects were used to account for repeated measurements within individuals. Adjusted least squares means (LSmeans) of food group intakes and sustainability indicators (accounting for sex, age, and total energy intake) were estimated from these models for each trajectory profile at three time points: 2014, 2018, and 2022. To enable comparisons across profiles, percentage variations for each food group and indicator at baseline were computed relative to the reference profile (P0, as defined in the Results section). Additionally, percentage variations within each profile were calculated to assess changes in food group consumption and sustainability indicators over the study period (2014–2022).

Health risk assessment was performed using the Comparative Risk Assessment (CRA) framework through the EpiDiet (Evaluate the Potential Impact of a Diet) model [44]. This approach enabled the assessment of the impact of dietary trajectories on health outcomes across profiles from 2014 to 2022, measured through DALYs avoided over time, while also examining the contributions of specific food groups to these modeled changes in health outcomes. Input data included the consumption of various food groups (fruits, vegetables, whole grains, nuts, seeds, milk, red meats, processed meats, sugar-sweetened beverages, legumes) and energy intake (means and standard deviations). The list of diet-related chronic diseases was derived from the 2017 Global Burden of Disease Study [45] and categorized using the 10th revision of the International Statistical Classification of Diseases and Related Health Problems [46].

DALYs combine two components: Years of Life Lost (YLL), reflecting premature mortality, and Years Lived with Disability (YLD), reflecting morbidity. YLLs were calculated as the number of premature deaths from diet-related chronic diseases multiplied by the standard life expectancy at the age of death, using age- and sex-specific mortality data from the Global Burden of Disease (GBD) Study 2017. YLDs were estimated by applying age-, sex-, and disease-specific conversion rates from GBD 2017, which represent the average ratio of YLD to YLL for each disease. This method allows for the estimation of DALYs at the population level without requiring individual-level data on disease duration or disability weights.

Point estimates of DALYs avoided for each profile were assessed in 2014 and 2022, relative to the reference scenario (whole sample in 2014). The change over time (2014–2022) was calculated by subtracting the baseline value (2014 compared to the whole sample in 2014) from the endpoint value (2022 compared to whole sample in 2014) within each profile. A positive value for DALYs avoided indicates a lower health risk for the profile, while a negative value indicates a higher health risk. To enable comparisons between profiles, percentage variations in total DALYs avoided were calculated at baseline and over time, as previously described. Decomposition Based on Absolute Contributions was applied to express the contributions of food groups to DALYs avoided as percentages, normalizing both positive and negative values relative to the total absolute sum within each profile.

The detailed methodology is provided in the Supplemental Method 5.

Data management and statistical analyses were performed using SAS software, Version 9.4 (SAS Institute Inc., Cary, NC, USA) and RStudio software (RStudio, Version 1.4.1717, © 2009–2021 RStudio, PBC).

## Results

### Characterization and description of dietary consumption trajectory profiles

Six dietary consumption trajectory profiles (P) were identified. The characteristics of the participants in the 6 profiles are shown in Table 1. The detailed values of food group consumption across profiles are presented in Table 2, and Fig. 1.

**Table 1:** Participants’ characteristics in the whole sample and in the dietary trajectory profiles (2014), n = 17,187, NutriNet-Santé Study^1,2^

	**Whole Sample**	**P0**	**P1**	**P2**	**P3**	**P4**	**P5**	**P**
Weighted n	n = 17,187	5,976	970	2,588	1,565	2,898	3,190	
Sex (%)								
Males	47.64	44.15	86.75	45.06	40.01	17.45	75.56	
Females	52.36	55.85	13.25	54.94	59.99	82.55	24.44	
Age (year)	48.38 (15.97)	48.22 (14.79)	60.52 (10.00)	40.61 (17.08)	40.93 (16.71)	60.93 (9.30)	43.55 (19.72)	< 0.0001
Occupational position (%)								< 0.0001
Self-employed/farmer	4.45	2.34	15.97	4.74	2.70	5.32	4.73	
Managerial staff/intellectual profession	9.10	11.93	5.42	8.27	10.01	4.75	9.11	
Unemployed	8.85	7.37	2.84	9.10	23.24	5.15	9.53	
Employee. manual worker	31.19	28.56	13.15	42.66	25.91	16.83	47.87	
Students	4.49	4.74	0	10.51	7.65	1.15	2.05	
Intermediate professions	14.50	19.02	7.14	15.09	13.34	9.48	12.89	
Retired	27.42	26.03	55.48	9.63	17.15	57.32	13.83	
Monthly income per household unit (%)								< 0.0001
Refuse to declare	6.58	5.78	3.95	4.90	8.33	8.43	7.70	
< 1200€/C.U	24.42	20.85	7.22	35.94	35.96	14.86	30.02	
1200—1800€/C.U	30.91	34.91	26.35	28.26	23.62	27.70	33.45	
1800—2700€/C.U	23.39	22.07	40.89	20.26	19.80	30.01	18.84	
> 2700€/C.U	14.70	16.39	21.60	10.64	12.30	18.99	10.00	
Place of residence (%)								< 0.0001
Rural community	23.99	23.27	29.06	22.81	15.03	25.02	28.19	
Urban unit (< 20,000 inhabitants)	18.63	18.88	16.52	18.11	17.57	16.19	21.93	
Urban unit (20,000 to 200,000 inhabitants)	16.80	16.48	13.27	12.1	15.64	20.52	19.48	
Urban unit (> 200,000 inhabitants)	40.58	41.37	41.16	46.97	51.74	38.26	30.38	
Smoking habits (%)								< 0.0001
Never smoker	50.16	53.16	24.24	58.63	61.13	50.53	39.81	
Former smoker	39.02	37.46	51.89	31.53	32.48	43.78	42.98	
Current smoker	10.83	9.38	23.88	9.85	6.39	5.70	17.21	
Physical activity (%)								< 0.0001
Low	20.30	22.43	29.16	21.55	9.27	11.62	25.91	
Moderate	31.16	30.96	24.35	41.36	33.74	29.79	25.32	
High	32.89	31.56	36.23	23.87	46.68	44.67	24.19	
Missing data	15.65	15.05	10.25	13.22	10.30	13.92	24.58	
BMI (kg/m^2^)	24.94 (4.99)	24.66 (4.45)	26.37 (3.70)	24.49 (5.50)	21.73 (3.19)	24.68 (3.44)	27.21 (9.10)	0.068

*Abbreviations:* *BMI* body mass index, *C.U* consumption unit;

^1^Values are mean (SD) or % as appropriate, all data are weighted

^2^P values were calculated using ANOVA or Chi^2^ test

**Table 2:** Food group consumption (g/d) over time (2014–2022) by profile, *n* = 17,187, NutriNet-Santé Study^1,2^

Food groups	P0		P1		P2		P3		P4		P5	
	**2014**	**2022**	**2014**	**2022**	**2014**	**2022**	**2014**	**2022**	**2014**	**2022**	**2014**	**2022**
Alcohol	101 (97;104)	95 (91;98)	404 (396;412)	394 (385;402)	70 (64;76)	70 (64;76)	68 (62;74)	75 (68;82)	93 (89;97)	81 (76;86)	109 (104;115)	105 (99;112)
*P* value	ref	ref	< 0.0001	0.26	< 0.0001	0.05	< 0.0001	0.0001	0.004	0.01	0.008	0.46
Butter	6 (6;7)	8 (7;8)	10 (9;10)	14 (13;14)	6 (6;6)	7 (7;8)	2 (2;3)	2 (1;2)	4 (3;4)	5 (5;6)	6 (6;6)	7 (6;7)
*P* value	ref	ref	< 0.0001	< 0.0001	0.003	0.52	< 0.0001	< 0.0001	< 0.0001	0.0007	0.10	0.0009
Dairy products	181 (177;185)	182 (177;186)	136 (127;145)	130 (119;141)	182 (175;188)	180 (173;188)	104 (97;112)	79 (70;87)	230 (225;235)	201 (195;207)	187 (180;193)	148 (141;155)
*P* value	ref	ref	< 0.0001	0.22	0.80	0.59	< 0.0001	< 0.0001	< 0.0001	< 0.0001	0.12	< 0.0001
Eggs	10 (10;11)	16 (15;16)	9 (8;10)	12 (11;14)	10 (9;11)	12 (11;13)	10 (10;11)	27 (26;28)	11 (11;12)	18 (17;18)	11 (11;12)	14 (13;15)
*P* value	ref	ref	0.003	0.02	0.38	< 0.0001	0.66	< 0.0001	0.04	0.0001	0.03	< 0.0001
Fish	44 (43;45)	42 (40;43)	45 (42;47)	45 (42;48)	35 (33;36)	34 (31;36)	46 (44;48)	37 (35;40)	61 (59;62)	64 (62;65)	44 (42;46)	58 (55;60)
*P* value	ref	ref	0.59	0.13	< 0.0001	0.38	0.05	< 0.0001	< 0.0001	< 0.0001	0.98	< 0.0001
Fruit juices	80 (77;83)	38 (34;41)	89 (82;96)	72 (64;80)	126 (121;131)	115 (109;121)	115 (109;120)	−12 (−18;−5)	85 (81;89)	38 (33;43)	47 (42;52)	38 (33;44)
*P* value	ref	ref	0.02	< 0.0001	< 0.0001	< 0.0001	< 0.0001	< 0.0001	0.04	0.12	< 0.0001	< 0.0001
Fruits	245 (239;252)	267 (259;274)	193 (177;209)	184 (166;203)	247 (236;259)	194 (181;208)	349 (336;362)	399 (385;414)	427 (418;436)	419 (408;430)	191 (180;202)	198 (186;210)
*P* value	ref	ref	< 0.0001	0.001	0.72	< 0.0001	< 0.0001	0.0002	< 0.0001	< 0.0001	< 0.0001	0.02
Hot drinks	761 (747;774)	805 (790;820)	779 (747;811)	808 (772;845)	548 (525;572)	616 (589;643)	738 (711;764)	780 (751;809)	916 (898;933)	964 (944;985)	678 (655;701)	758 (733;783)
*P* value	ref	ref	0.31	0.37	< 0.0001	0.06	0.12	0.86	< 0.0001	0.72	< 0.0001	0.001
Legumes	13 (12;14)	18 (17;19)	9 (7;11)	14 (11;16)	6 (4;7)	10 (9;12)	41 (39;42)	53 (51;55)	17 (16;18)	20 (19;22)	11 (10;13)	20 (18;21)
*P* value	ref	ref	0.0002	0.91	< 0.0001	0.79	< 0.0001	< 0.0001	< 0.0001	0.09	0.02	0.0001
Milk	60 (56;64)	33 (28;37)	18 (9;27)	12 (1;22)	172 (166;179)	151 (143;159)	23 (15;30)	19 (10;27)	58 (53;63)	48 (41;54)	38 (32;44)	69 (62;76)
*P* value	ref	ref	< 0.0001	0.0002	< 0.0001	0.12	< 0.0001	< 0.0001	0.54	< 0.0001	< 0.0001	< 0.0001
Nuts	4 (3;4)	10 (9;10)	4 (3;5)	6 (5;7)	2 (1;3)	8 (7;9)	30 (29;31)	42 (41;43)	12 (12;13)	20 (19;20)	2 (2;3)	5 (4;6)
*P* value	ref	ref	0.97	< 0.0001	< 0.0001	0.33	< 0.0001	< 0.0001	< 0.0001	< 0.0001	0.0004	< 0.0001
Offal	4 (4;5)	4 (3;4)	16 (15;17)	1 (0;2)	4 (3;5)	3 (2;4)	1 (1;2)	2 (1;2)	4 (4;5)	3 (3;4)	7 (6;7)	6 (5;7)
*P* value	ref	ref	< 0.0001	< 0.0001	0.31	0.69	< 0.0001	0.16	0.61	0.72	< 0.0001	0.97
Oil	30 (29;30)	35 (34;35)	31 (29;32)	34 (32;35)	19 (18;20)	24 (22;25)	30 (29;31)	40 (38;41)	30 (29;31)	34 (33;35)	24 (24;25)	33 (32;34)
*P* value	ref	ref	0.24	0.01	< 0.0001	0.62	0.75	< 0.0001	0.53	0.05	< 0.0001	< 0.0001
PMD	43 (−396;482)	33 (−406;472)	31 (−1017;1078)	40 (−1007;1088)	100 (−747;948)	110 (−738;957)	23 (−827;873)	29 (−821;879)	29 (−528;586)	33 (−524;590)	33 (−815;881)	34 (−814;882)
*P* value	ref	ref	0.98	< 0.0001	0.90	< 0.0001	0.97	< 0.0001	0.97	< 0.0001	0.98	< 0.0001
Plant-based substitutes	11 (9;14)	21 (18;24)	2 (−4;8)	5 (−3;12)	−2 (−7;2)	39 (33;44)	164 (158;169)	155 (149;161)	26 (22;29)	45 (40;49)	4 (0;9)	7 (2;12)
*P* value	ref	ref	0.01	0.10	< 0.0001	< 0.0001	< 0.0001	< 0.0001	< 0.0001	0.0005	0.008	0.01
Pork	15 (14;15)	14 (14;15)	19 (18;20)	18 (17;19)	14 (14;15)	9 (8;10)	4 (3;4)	5 (4;6)	9 (8;9)	7 (6;8)	36 (35;36)	33 (32;34)
*P* value	ref	ref	< 0.0001	0.09	0.56	< 0.0001	< 0.0001	0.0001	< 0.0001	0.0001	< 0.0001	< 0.0001
Potatoes	29 (28;29)	28 (27;28)	28 (26;29)	30 (28;32)	22 (21;23)	24 (22;25)	21 (20;22)	29 (27;30)	18 (18;19)	21 (19;22)	40 (39;41)	36 (34;37)
*P* value	ref	ref	0.19	< 0.0001	< 0.0001	< 0.0001	< 0.0001	< 0.0001	< 0.0001	< 0.0001	< 0.0001	< 0.0001
Poultry	25 (24;26)	23 (23;24)	21 (19;23)	21 (19;23)	26 (25;27)	18 (17;20)	10 (8;11)	5 (3;6)	21 (20;22)	19 (17;20)	50 (49;51)	46 (45;47)
*P* value	ref	ref	< 0.0001	0.08	0.18	< 0.0001	< 0.0001	0.0001	< 0.0001	0.11	< 0.0001	0.002
Processed meat	23 (22;24)	30 (29;30)	23 (21;24)	40 (38;41)	22 (21;23)	28 (27;30)	8 (7;10)	7 (6;8)	16 (15;17)	20 (19;21)	38 (37;39)	53 (52;54)
*P* value	ref	ref	0.78	< 0.0001	0.06	0.79	< 0.0001	< 0.0001	< 0.0001	< 0.0001	< 0.0001	< 0.0001
Ruminant meat	41 (40;42)	33 (32;34)	53 (50;55)	42 (39;44)	40 (39;42)	35 (33;36)	12 (10;13)	13 (11;15)	28 (27;29)	24 (23;25)	78 (76;79)	61 (59;63)
*P* value	ref	ref	< 0.0001	0.06	0.48	0.01	< 0.0001	< 0.0001	< 0.0001	< 0.0001	< 0.0001	< 0.0001
Refined cereals	157 (155;160)	126 (123;128)	113 (107;118)	84 (78;90)	126 (122;130)	104 (100;109)	66 (62;71)	66 (61;71)	94 (91;97)	74 (70;78)	158 (154;162)	145 (140;149)
*P* value	ref	ref	< 0.0001	0.36	< 0.0001	< 0.0001	< 0.0001	< 0.0001	< 0.0001	< 0.0001	0.77	< 0.0001
SSFF	86 (84;87)	83 (82;85)	66 (62;69)	81 (77;85)	121 (119;124)	119 (116;122)	59 (56;62)	56 (53;60)	66 (64;68)	68 (65;70)	74 (71;76)	74 (72;77)
*P* value	ref	ref	< 0.0001	< 0.0001	< 0.0001	0.77	< 0.0001	0.94	< 0.0001	0.006	< 0.0001	0.02
SSB	37 (34;40)	30 (27;33)	32 (25;39)	28 (20;37)	131 (126;136)	64 (58;70)	26 (21;32)	7 (1;14)	25 (21;29)	22 (17;27)	39 (35;44)	41 (36;47)
*P* value	ref	ref	0.19	0.39	< 0.0001	< 0.0001	0.0006	0.0003	< 0.0001	0.11	0.44	0.0004
Vegetables	323 (317;329)	336 (329;342)	286 (272;299)	300 (284;317)	221 (211;231)	302 (291;314)	453 (442;464)	590 (577;603)	445 (437;452)	456 (447;465)	288 (279;298)	313 (302;324)
*P* value	ref	ref	< 0.0001	0.78	< 0.0001	< 0.0001	< 0.0001	< 0.0001	< 0.0001	0.78	< 0.0001	0.02
Whole-grain products	46 (44;48)	78 (75;80)	45 (41;50)	58 (53;64)	19 (15;22)	45 (40;49)	173 (169;177)	109 (104;113)	96 (93;99)	94 (91;98)	34 (30;37)	42 (38;46)
*P* value	ref	ref	0.73	< 0.0001	< 0.0001	0.02	< 0.0001	< 0.0001	< 0.0001	< 0.0001	< 0.0001	< 0.0001

*Abbreviations:* *SSFF* Salty or sweet and Fatty Foods, *SSB* Sugar-sweetened beverages, *PMD* Prepared and Mixed Dishes

^1^Values are the least squares means (CI) of adjusted consumptions (for sex, age, and total energy intake)

^2^P-values are those of β-coefficients of the fixed-effects models. The p-values in 2014 indicate the significance of the difference in baseline consumption level compared to the reference profile (P0). The p-values in 2022 indicate the significance of the difference in consumption variation over time (2014–2022) compared to the difference in reference profile (P0)

Profile P0 (35% of the population, 56% women, average age = 48 y (SD = 15)) was the largest group, with socioeconomic characteristics similar to those of the whole sample. This profile was characterized by baseline consumption levels close to the population average for nearly all food groups. Given its sociodemographic similarity to the overall population and its alignment with the consumption levels previously observed in the whole sample [19], P0 was chosen as the reference trajectory. Over time (2014–2022), individuals within this profile showed statistically significant increases in the consumption of plant-based substitutes (a 85% increase), wholegrain products (+ 67%), fruits (+ 9%), hot drinks (+ 6%) and vegetables (+ 4%), Conversely, their consumption of ruminant meat (−20%) decreased over the same period, as did their intake of fruit juices (−53%), milk (−46%), refined cereals (−20%), and SSB (−19%).

All changes in food group consumption between baseline and 2022 were statistically significant within each profile (p < 0.05), except for prepared and mixed dishes (PMD), for which no significant change was observed.

Profile P1 (6% of the population, 87% men, average age = 61y (SD = 10)) had the highest baseline consumption of alcohol (+ 301%), butter (+ 48%), and offal (+ 281%), along with elevated intakes of pork (+ 32%) and ruminant meat (+ 29%) compared to P0. Over time (2014–2022), and compared to changes observed in the other profiles, this profile showed the greatest increase in the consumption of processed meat (40 g/day in 2022; + 74% compared to 2014), PMD (40 g/day; + 31%), and SSFF (80 g/day; + 23%), along with the most substantial decrease in offal (1 g/day; −95%). All these changes were statistically significant.

Profile P2 (15% of the population, 55% of women, average age = 41y (SD = 17)) was characterized by the highest proportion of low-income individuals (68% with income < 1800€/C.U.) and a notable proportion of students (10%). This profile had significantly higher baseline consumption of SSB (+ 251%), milk (+ 187%), fruit juices (+ 58%), and SSFF (+ 41%) compared to P0. Over time, compared to 2014, this profile exhibited the sharpest increase in the consumption of plant-based substitutes (39 g/day in 2022; + 1737% compared to 2014), wholegrain products (45 g/day; + 139%) and vegetables (302 g/day; + 37%), alongside marked decreases in SSB (64 g/day; −51%), and fruits (194 g/day; −21%).

Profile P3 (9% of the population, 60% of women, average age = 41 (SD = (17)) had higher baseline consumption of plant-based substitutes (+ 1364%), nuts (+ 680%), wholegrain products (273%), legumes (+ 205%), and vegetables (+ 40%) compared to P0. Over time, compared to 2014, this profile significantly increased their consumption of eggs (27 g/day in 2022; + 166% compared to 2014), ruminant meat (13 g/day; + 13%) and alcohol (75 g/day; + 10%), along with the most pronounced decrease in wholegrain products (109 g/day; −37%), fish (37 g/day; −20%), and plant-based substitutes (155 g/day; −5%).

Profile P4 (17% of the population, 83% of women, average age = 61y (SD = 9)) included individuals with higher income levels (19% with income > 2700€/C.U.) and high levels of physical activity (46%). It was characterized by the highest baseline consumption of fruit (+ 74%), fish (+ 39%), dairy products (+ 27%), and also had a high consumption of nuts (+ 212%), plant-based substitutes (+ 131%), wholegrain products (+ 107%), vegetables (+ 38%) and legumes (+ 29%) compared to P0. Over time, compared to 2014, this profile showed a significant increase in plant-based substitutes (45 g/day in 2022; + 73% compared to 2014), and vegetables (456 g/day; + 3%), and the most pronounced decrease in alcohol (81 g/day; −13%).

Profile P5 (19% of the population, 76% of men, average age = 44y (SD = 19)) included the highest proportion of individuals living with obesity (26%). At baseline, they had the highest consumption of pork (+ 143%), poultry (+ 98%), ruminant meat (+ 90%), processed meat (+ 66%), and potatoes (+ 39%) compared to P0. Over time, compared to 2014, this profile demonstrated the most substantial rise in the consumption of milk (69 g/day in 2022; + 82% compared to 2014), and fish (58 g/day; + 31%), as well as an increase in legumes (20 g/day; + 72%), wholegrain products (42 g/day; + 24%), and vegetables (313 g/d; + 9%). A marked decrease was observed for dairy products (148 g/day; −21%).

### Multicriteria analysis of profiles’ sustainability

**Fig. 2:**
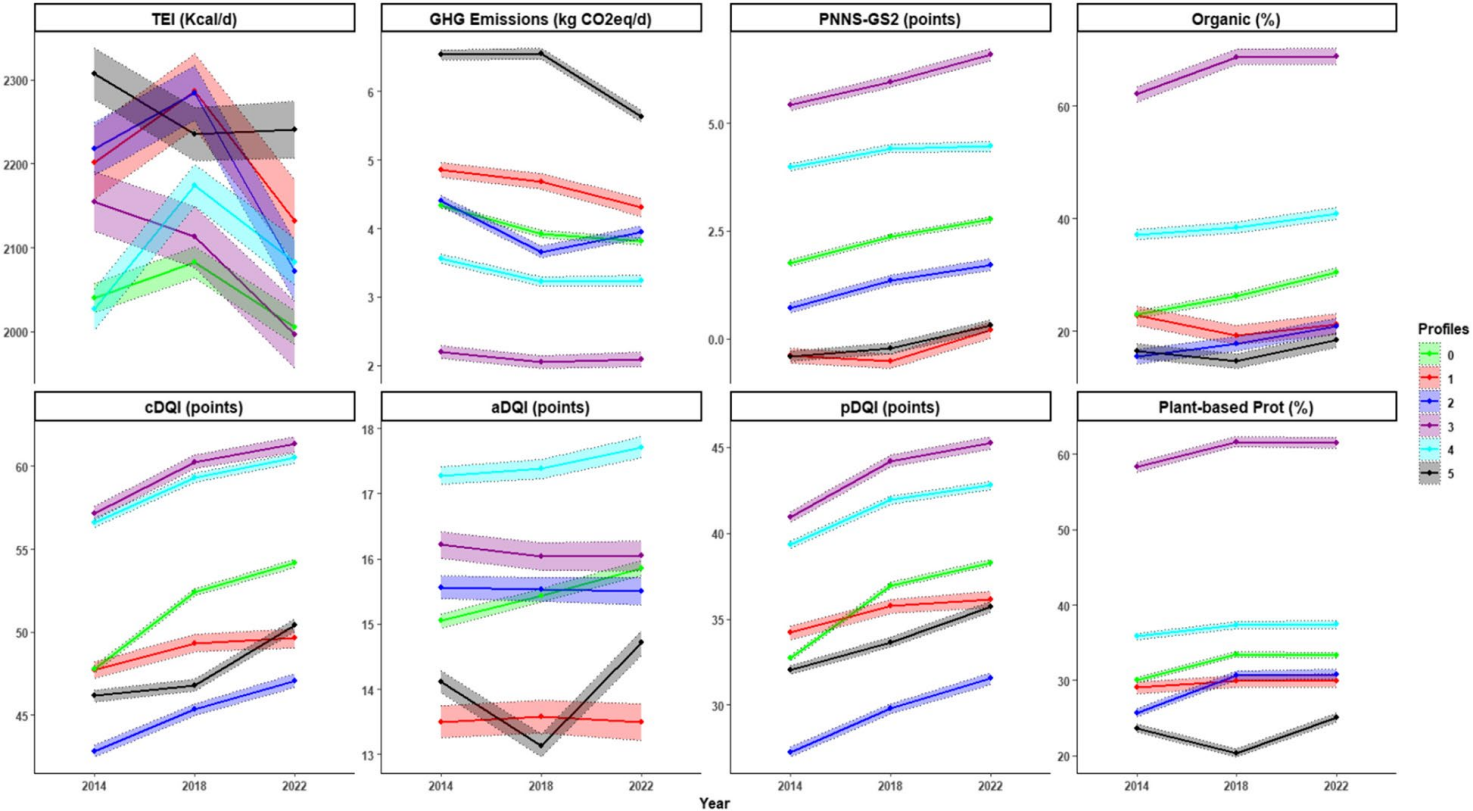
Evolution trajectories for sustainability indicators (2014 to 2022) by profile, n = 17,187, NutriNet-Santé Study^*1,2*^ . Abbreviations: TEI, Total Energy Intake (Kcal/d); GHG, Greenhouse Gas (kg CO2eq/d); PNNS-GS2: Programme National Nutrition Santé-Guidelines Score 2 (adherence to French dietary guidelines score); cDQI, Comprehensive Diet Quality Index; pDQI, plant-based Diet Quality Index; aDQI, animal-based Diet Quality Index; Plant-based Prot, Plant-based Proteins. ^1^For all indicators, values are the least squares means (CI) of adjusted scores (for sex, age, and total energy intake). ^2^The x-axis represents time (2014; 2018; 2022), while the y-axis represents indicator scores.

**Fig. 3:**
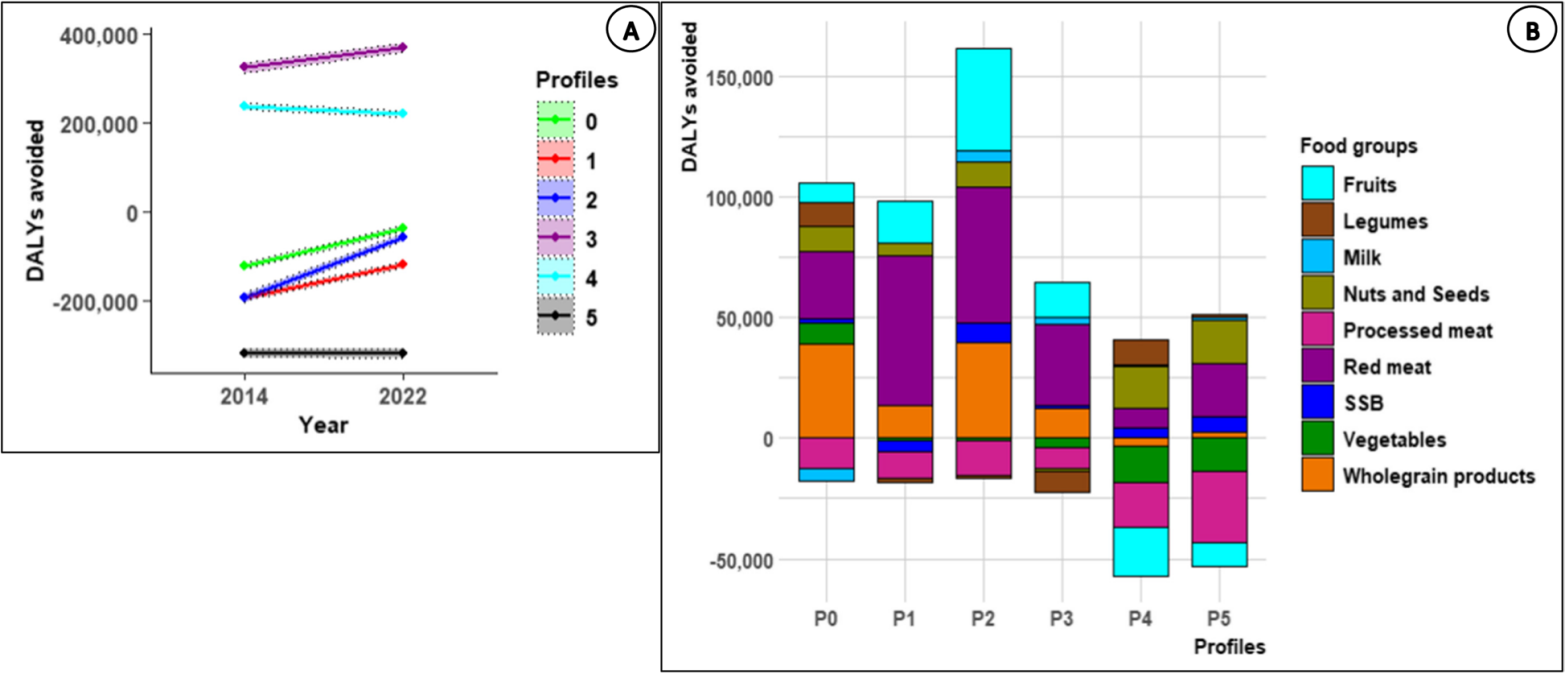
DALYs avoided over time (2014 to 2022) by profile (A)^1^; Food group contributions to the variation (2014–2022) of DALYs avoided by profile (B)^2^ . Abbreviations: SSB, Sugar-Sweetened Beverages. The Red meat food group encompasses beef, lamb, pork, mutton, and goat ^1^(A) Positive values for DALYs avoided indicate a reduction in health risk for the profile compared to the whole sample in 2014. Negative values indicate an increase in health risk for the profile relative to the whole sample in 2014. ^2^(B) Positive values reflect a reduction in health risk (increase in DALYs avoided) over time (2014–2022) due to changes in food group consumption (increase in healthy foods and decrease in unhealthy foods) for the profile. Negative values reflect an increase in health risk (decrease in DALYs avoided) over time (2014–2022) due to changes in food group consumption (increase in unhealthy foods and decrease in healthy foods) for the profile

Results of the multicriteria analysis of profiles are presented in Fig. 2 (evolution of sustainability indicators), and Fig. 3 (DALYs avoided and food group contributions) as well as Supplemental Table 1 (sustainability indicators over time).

a Total energy intake
  P5 had the highest TEI at baseline (+ 13%), followed by P2 (+ 9%), P1 (+ 8%), and P3 (+ 6%) compared to the reference profile P0 (2040 kcal/day). P4, which had the lowest baseline TEI (2027 kcal/day), was the only profile that did not exhibit a decline in TEI over time. Detailed values are presented in Supplemental Table 1 and Fig. 2.
b Dietary quality
  At baseline, P3 (+ 20%) and P4 (+ 19%) had the highest cDQI scores, while P2 recorded the lowest (−10%) compared to the reference profile P0 (47.78 points). Over time (2022 compared to 2014), cDQI improved the most for P0 (+ 13%), followed by P2 (+ 10%) and P5 (+ 9%), with smaller increases for P3 and P4 (+ 7%) and the smallest for P1 (+ 4%). The greatest improvements were observed in the pDQI component.
  Regarding adherence to French food-based dietary guidelines, the highest baseline PNNS-GS2 was observed in profiles P3 (+ 208%) and P4 (+ 126%) compared to P0 (1.76 points). Profiles P5 (−123%), P1 (−122%), and P2 (−59%) exhibited the lowest adherence to nutritional guidelines compared to P0. Over time, the whole population showed improvements in PNNS-GS2. The most substantial increases were observed in profiles P5 (+ 174%), P1 (+ 151%), and P2 (+ 139%), while more modest increases were observed in profiles P0 (+ 57%), P3 (+ 22%), and P4 (+ 12%).
  Initially higher in profiles P3 (+ 94%) and P4 (+ 19%), compared to P0 (30% of total protein intake), plant-based protein intake increased across all profiles at nearly similar rates. Detailed values are presented in Supplemental Table 1 and Fig. 2.
c Health risk
  At baseline, P3 and P4 achieved the highest DALYs avoided compared to P0 (+ 363% and + 292%, respectively), reflecting the lowest health risks. In contrast, P5 had the highest health risk, with 159% fewer DALYs avoided than P0. Between 2014 and 2022, P2 showed the greatest increase in DALYs avoided (+ 70%), followed by P0 and P1 (+ 70% and + 39%). Besides, P4 and P5 exhibited a decline in DALYs avoided (−7% and −0.5%, respectively). The contributions of food groups to DALYs avoided showed that the reduction in health risk over time (2014–2022) was mainly driven by decreased red meat consumption, contributing 8% (P4) to 53% (P1) to the DALYs avoided. The increase in fruit and wholegrain products consumption positively contributed to DALYs avoided in profiles P0, P1, P2, and P3, with fruits accounting for 7% (P0) to 24% (P2) and wholegrain products for 12% (P1) to 32% (P0). Conversely, the rise in health risk in profiles P4 and P5 was largely due to increased processed meat consumption, which negatively affected DALYs avoided by −28% (P5) and −19% (P4). These increases in health risk were also driven by reduced fruit consumption, contributing −24% (P4) and −9% (P5) to DALYs avoided, and reduced vegetable consumption, contributing −16% (P4) and −13% (P5) to DALYs avoided. Detailed values are presented in Supplemental Tables 1 and 2, and Fig. 3.
  It should be noted that trends for certain food groups may differ slightly depending on the statistical approach used. While trajectory analyses based on mixed models account for covariates such as age, sex, and repeated measures, the health risk assessment relies on simple energy-adjusted means using the residual method. These methodological differences can lead to small variations in observed trends across food groups and should be taken into consideration when interpreting the results.
d Greenhouse gas emissions
  At baseline, participants in the P0 profile showed food-related GHG emissions of 4.33 kg of CO_2_eq/d. Compared to P0, the highest emissions were observed in P5 (+ 51%) and P1 (+ 12%), while the lowest were observed in P3 (−49%) and P4 (−18%). Emissions in P2 were similar to those of P0 (+ 1%).
  Over the period from 2014 to 2022, GHG emissions for the reference profile P0 decreased by 12%. The most significant reduction was observed in P5 (−14%). Reductions in P1 (−11%), P2 (−10%), and P4 (−9%) were comparable to those in P0, whereas P3 showed the smallest reduction (−5%). Detailed values are presented in Supplemental Table 1 and Fig. 2.
e Organic food consumption across profiles
  At baseline, the percentage of organic foods in the diet was 23% for P0. It was notably higher for P3 (+ 170% compared to P0), followed by P4 (+ 62% compared to P0). In contrast, profiles P2 (−32%) and P5 (−28%) had lower consumption of organic products compared to P0, while P1 (−1%) had levels similar to P0. Over time (2014–2022), organic consumption increased in profile P2 (+ 35%) at a rate similar to that of the reference profile P0 (+ 33%). Increases were also observed in profiles P5 (+ 12%), P3 (+ 11%), and P4 (+ 10%), whereas P1 experienced a slight decrease (−7%) over time. Detailed values are presented in Supplemental Table 1 and Fig. 2.

## Discussion

Our study identified six distinct dietary trajectory profiles, each reflecting unique starting diets and changes over time. One profile exhibited average intakes (P0), while others displayed more specific dietary trajectories: two profiles, predominantly male (P1 and P5), were more meat-focused, with one (P1) also associated with high alcohol consumption and the other (P5) showing the highest intake of animal products. In contrast, two other profiles (P3 and P4) leaned toward plant-based diets, with one, primarily composed of females (P4), maintaining high intakes of fish and plant-based foods, while the other (P3) showed an increase in ruminant meat consumption over time. A profile with typically lower income levels (P2) was notable for its high intake of salty or sweet fatty foods but also showed the most significant shift toward plant-based foods. These dietary trajectories revealed improvements in some sustainability indicators, though the extent of these varied widely across the profiles.

To the best of our knowledge, longitudinal studies on observed dietary consumption trajectories, especially those using recent data, remain limited [16–18]. The dominant profile (P0), considered as the reference profile with a sociodemographic structure comparable to the general population, exhibited an upward trend in the consumption of plant-based foods, concomitantly with reductions in ruminant meat and unhealthy plant-based food intake. This shift toward more plant-based diets aligns with trends observed across the WHO European Region [47]. This transition led to improved dietary quality, similar to the positive changes reported in the Netherlands between 1993 and 2015, and better adherence to dietary guidelines [16]. In contrast, a Swedish study highlighted a deviation from Nordic dietary recommendations among participants between 2008 and 2016 compared to 2000–2007, reflecting less favorable dietary shifts [17]. While the dominant profile (P0) trajectory is consistent with these global studies, our research aims to identify profile disparities, as these variations highlight distinct dietary patterns, with differing sustainability impacts. To explore these distinctions, we interpreted the trajectories through the lens of behavioral typologies and assessed their related sustainability outcomes.

The highest adherence to dietary guidelines levels were found in the profiles with predominantly plant-based diets (P3 and P4), while the lowest were observed in the profiles with high meat consumption (*P5* and *P1*). Extensive evidence underscores the significant long-term benefits of plant-based diets, highlighting their protective role against chronic disease and overall mortality [48, 49]. In line with this, increased fruit and wholegrain consumption were key contributors to reducing health risks, as reflected in higher DALYs avoided in profiles P0, P1, P2, and P3. Profile P2 showed the greatest improvement, largely due to its significant shift toward plant-based foods. In contrast, reduced fruit and vegetable intake and a substantial rise in processed meat consumption were major contributors to fewer DALYs avoided over time in profiles P4 and P5, despite P4 initially adhering to a plant-based diet. However, in absolute terms, the DALYs avoided by P4 in 2022 remain considerably higher than those of profiles P0, P1, P2, and P5, although the highest value was observed for P3. Notably, P4 was predominantly composed of women, and participants with a healthy lifestyle (46% engaging in vigorous physical activity, 94% being never-smokers).

In terms of sex differences, the existing literature highlights sex as a significant determinant of dietary consumption [50–53], with women consuming more legumes and wholegrain products [50, 54] and being more likely to follow the recommendation of five daily servings of fruits and vegetables [50, 53]. In contrast, while some improvement was observed over time, the predominantly male profile (P1) consistently displayed the lowest adherence to dietary guidelines throughout the study period. This finding aligns with research indicating that men tend to consume higher amounts of high-fat foods, high-protein foods [50, 51] and meat [55, 56]. Nevertheless, the improvement in dietary quality within this profile may partly be attributed to older baseline age (over 60 years), as studies have shown that dietary quality generally improves with age and tends to be higher among individuals over 60, likely due to the onset of co-morbidities [29, 57–59]. Additionally, the profile showing the most significant increase in plant-based food consumption (P2) consisted of 64% of individuals with low to moderate incomes and a high proportion of students. Despite this notable shift, P2 started with the lowest vegetable consumption among all profiles and also experienced a decline in fish consumption over time, which was already low at baseline. While adherence to dietary guidelines and overall diet quality improved in this profile, it remained lower than that of the reference profile (P0). This finding aligns with existing literature, which often associates higher income levels with better diet quality in adults [57, 60]. Conversely, profile P4, characterized predominantly by individuals with moderate to high incomes at baseline, exhibited the highest absolute consumption of fish and fruit, as well as the highest aDQI score. Thus, our findings highlight the critical role of targeting key food groups (red meat, processed meat, fruits, and wholegrain products) as key contributors to health outcomes in dietary transitions. But it is also crucial to align dietary transition strategies with the specific dietary patterns and nutritional needs of each profile to optimize health outcomes and more effectively support the health dimension of sustainable dietary shifts.

In line with previous research on the associations between energy intake, meat consumption, and greenhouse gas (GHG) emissions [61–63], our findings confirm that the profile with the highest energy intake (P5) also had the highest meat consumption and was associated with significantly greater dietary GHG emissions. In contrast, the profile with the lowest energy intake (P4) was characterized by a higher consumption of plant-based foods and much lower emissions. These results are consistent with prior studies indicating that reducing meat intake, particularly ruminant meat such as beef, is a key lever for lowering the carbon footprint of diets at the population level [64–66]. Additionally, modeling studies suggest that increasing the proportion of plant-based proteins in the diet could lead to substantial reductions in GHG emissions, with potential decreases of up to 70% when shifting toward healthier and more plant-based dietary patterns [67–70]. Taken together, our findings underscore the pivotal role of shifting from animal-based to plant-based proteins in improving dietary environmental sustainability [34] and support the need to address meat consumption patterns to reduce environmental impacts. Also, in our study, the profiles with the highest GHG emissions (P5 and P1) were predominantly composed of males. This is consistent with evidence suggesting that GHG emissions in men are approximately 30% higher than in women [71], and up to 41% higher in another study [72]. Furthermore, alcohol consumption, which is more prevalent in the predominantly male profile P1, contributes to food-related GHG emissions. According to the literature, alcohol typically accounts for around 3% of total food-related GHG emissions [73]. Still, in populations with higher alcohol intake, this can range from 6 to 11%, with GHG emissions being 90% higher in men [73].

We also observed an increase in the contribution of organic food across nearly all profiles, reflecting a broader trend in the French population, where the share of the food budget dedicated to organic products rose from 2.5% in 2014 [74] to 6% in 2022 [75], despite a slight decline between 2021 and 2022 [75]. Notably, 41% of food consumed by individuals in profile P4 was organic in 2022, despite inflationary pressures. The profile characterized by a predominantly plant-based diet (P3) consistently had the highest share of organic food consumption throughout the study period, aligning with their higher intake of plant-based foods, as reported in previous research [34, 76].

Although organic food share was considered here as a descriptive rather than analytical variable, it remains relevant for sustainability. Organic production generally avoids synthetic inputs, reduces chemical pollution, enhances biodiversity, and supports soil health and carbon sequestration, contributing to lower environmental impacts compared to conventional systems [77–79]. However, its overall contribution to sustainability depends on factors such as yields, land use, and dietary composition [77, 80]. In particular, organic consumption is most environmentally beneficial when combined with a largely plant-based diet [81].

Our findings revealed slight increases in the consumption of ruminant meat, pork, alcoholic beverages, and eggs within the predominantly plant-based profile (P3). While these changes remain minor in absolute terms, they could signal an early indicator of broader future dietary shifts, warranting future surveillance. Notably, P3's overall consumption levels for these items remained the lowest among all profiles, except for eggs, preserving its largely plant-based orientation. However, similar trends have been observed in studies showing that vegetarians and vegans are more likely to revert to omnivorous diets than the reverse [82]. Likewise, in Germany,"convinced flexitarians"increased their meat consumption due to waning belief in the benefits of plant-based diets [83]. These findings highlight the importance of targeted strategies to support and stabilize plant-based dietary behaviors over time, as small changes may signal the potential for broader future shifts.

### Strengths and limitations

Since participation in the NutriNet-Santé cohort is voluntary, our sample is not representative of the French population [84]. Participants tend to be more health-conscious, with a higher proportion of individuals in the healthy BMI range and a lower prevalence of obesity. Although we have addressed this limitation by applying data weighting to limit potential selection bias, caution is needed when generalizing. This health-conscious profile may partly explain the observed decrease in energy intake over time. Nevertheless, the paradox of declining reported energy intake alongside rising obesity rates in Western countries highlights the multifactorial nature of weight regulation, involving changes in physical activity, dietary composition, and broader environmental factors. Secondly, although under- and over-reporters are systematically excluded and validated dietary assessment tools are used, some degree of selective underreporting of specific foods may persist, as in all large-scale cohorts based on self-reported data. Nevertheless, the combination of repeated measures, rigorous quality controls, and energy adjustment procedures in NutriNet-Santé helps to minimize this bias. Moreover, LCA were restricted to the production stage since post-farm data were unavailable for the whole organic food system. While this means that our GHGe estimates are somewhat underestimated, the impact is likely limited, as production is by far the main cause of environmental pressures [8]. Another limitation of our approach is that focusing solely on GHG emissions does not capture the full spectrum of environmental impacts associated with food production, such as land use, water use, eutrophication, and biodiversity loss. This is particularly relevant when comparing organic and conventional production systems, which may differ more substantially in these other impact categories [85]. Nevertheless, GHG emissions remain a highly relevant and widely used indicator for assessing the environmental impact of diets, especially in the context of climate change mitigation [1, 26–28]. Future research could benefit from incorporating a broader range of sustainability metrics as data availability improves. Regarding health risk assessment, a limitation of this study is the use of GBD 2017 data, while more recent estimates (GBD 2021 [86]) are now available. Although the core diet–disease relationships remain well-established, future research should integrate GBD 2021 data to refine absolute DALY estimates, particularly in light of post-2019 trends affected by the COVID-19 pandemic and methodological updates. Furthermore, our DALY estimates rely on population-level parameters and conversion factors from the Global Burden of Disease Study 2017, as individual-level data on disease incidence, duration, and disability weights were not available. While this approach is widely used and enables international comparability, it introduces uncertainty, particularly regarding the absolute values of DALYs. Therefore, our results should be interpreted with caution, especially for absolute health impact estimates, while relative comparisons between profiles remain robust. That said, our study is one of the few that examines recent individual changes in dietary behaviors within a French cohort based on detailed, validated and consistently repeated dietary data collection over time covering a wide range of indicators related to sustainable diet. The large sample size allows us to explore a wide variety of profiles, including those who have undertaken transitions to sustainable dietary consumption. This provides a valuable opportunity to analyze their characteristics and draw insights from their dietary trajectories. In addition, studying different consumer profiles would allow a detailed analysis of the specific motivations and potential barriers to sustainable transitions in each segment, which is crucial for improving the effectiveness of targeted initiatives.

## Conclusions

This study reveals the varied dietary patterns of some French adults from 2014 to 2022, showcasing distinct eating profiles and trends. Our results indicate that moving towards more sustainable diets is achievable, as all these profiles exhibit improvements in some specificities of their diets, albeit to differing degrees, which in turn affect environmental, nutritional, and health outcomes. These distinctions highlight the necessity for customized strategies to meet the unique needs and attitudes of different consumer segments, alongside the importance of deepening our understanding of the socio-economic, psychological, and cultural factors that shape these dietary changes.

## Supplementary Information

Supplementary Material 1.

## Data Availability

Analytic code will be made available upon request. Researchers from public institutions can submit a collaboration request including information on their institution, funding sources and a brief description of the project to collaboration@etude-nutrinet-sante.fr. All requests will be reviewed by the steering committee of the NutriNet-Santé study. If the collaboration is accepted, a data access agreement will be necessary and appropriate authorizations from the competent administrative authorities may be needed. In accordance with existing regulations, no personal data will be accessible.

## Abbreviations

aDQI: Animal-based Diet Quality Index
C.U.: Consumption Unit
cDQI: Comprehensive Diet Quality Index
CRA: Comparative Risk Assessment
DALYs: Disability-Adjusted Life Years
FFQ: Food Frequency Questionnaire
GBMTM: Group-based multi-trajectory modeling
GHG: Greenhouse Gas
Org-FFQ: Organic Food Frequency Questionnaire
pDQI: Plant-based Diet Quality Index
PMD: Prepared and Mixed Dishes
PNNS-GS2: *Programme National Nutrition Santé*-Guidelines Score 2 (adherence to French dietary guidelines score)
SD: Standard deviation
SSFF: Salty and Sweetened Fatty Foods
SSB: Sugar-sweetened beverages
TEI: Total Energy Intake
