# Supplementary material for: Dietary consumption trajectory profiles over time of French adults from the NutriNet-Santé cohort (2014–2022): multicriteria analysis of sustainability
Source: Int J Behav Nutr Phys Act. 2025 Jun 13;22:76. doi: 10.1186/s12966-025-01777-w (PMC12166596; doi:10.1186/s12966-025-01777-w)
Supplement: Supplementary file 1 — Supplementary Material 1. [file 12966_2025_1777_MOESM1_ESM.docx]

**Dietary consumption trajectory profiles over time of French adults from the NutriNet-Santé cohort (2014-2022): Multicriteria analysis of sustainability**

Hafsa Toujgani^1^, Juhui Wang^2^, Elie Perraud^2^, Julia Baudry^1^, Justine Berlivet^1^, Benjamin Allès^1^, Hélène Fouillet^2^, Serge Hercberg^1^, Mathilde Touvier^1^, Denis Lairon^3^, Philippe Pointereau^4^, Christian Couturier^4^, François Mariotti^2^, Emmanuelle Kesse-Guyot^1^ and the TRANSFood Consortium

**Online Supplemental material**

**Table of Contents**

[Supplemental Method 1: Assessment of organic food group consumption 2](#_Toc187851245)

[Supplemental Method 2: Computation of the environmental indicators 3](#_Toc187851246)

[Supplemental Method 3: Computation of dietary indexes 4](#_Toc187851247)

[Supplemental Method 4: Group-based multi-trajectory modeling (GBMTM) method 5](#_Toc187851248)

[Supplemental Method 5: EpiDiet Simulation-Based Nutritional and Epidemiologic Model 6](#_Toc187851249)

[Supplemental Table 1: Sustainability indicators over time (2014-2022) by profile, n = 17,187, NutriNet-Santé Study 8](#_Toc187851250)

[Supplemental Table 2: Food group contributions to the variations (2014-2022) of DALYs avoided by profile, n = 17,187, NutriNet-Santé Study 9](#_Toc187851251)

[Supplemental Figure 1: Selection of the study sample 10](#_Toc187851252)

[References 11](#_Toc187851254)

## Supplemental Method 1: Assessment of organic food group consumption

Participants in the NutriNet-Santé Cohort were asked to report their consumption frequencies for 264 food and beverage items using a semi-quantitative food frequency questionnaire (FFQ). The organic food frequency questionnaire (Org-FFQ) was adapted from an original FFQ (1). For each item, except those unavailable under the organic label (e.g., water and sweeteners), a 5-point ordinal scale from “never” to “always” was used to assess the proportion of intake from organic sources. Participants had to respond to the question: “How often was the product of organic origin?”. For butter and margarine used for bread or frying, participants selected the most frequently consumed item from approximately 20 organic or conventional options. To estimate organic intake, weights of 0, 0.25, 0.5, 0.75, and 1 were assigned to the response options of never, rarely, half the time, often, and always, respectively. Sensitivity analyses were previously conducted to evaluate the impact of assigning arbitrary percentages, where 10% instead of 25% was assigned to the “rarely” category. Additionally, 20 Monte Carlo simulations (2) were performed, assigning arbitrary intervals to each frequency category: “never” (0% to 2.5%), “rarely” (2.5% to 35%), “half the time” (35% to 65%), “often” (65% to 90%), and “always” (90% to 100%). The modalities were assumed to be uniformly distributed within these intervals. Each dataset attributed the same percentage to a given modality. In those sensitivity analyses, the rankings of participants according to the share of organic food in the diet were similar.

## Supplemental Method 2: Computation of the environmental indicators

The analysis scope considered by the DIALECTE tool (3) is limited to the agricultural production stage. The production of inputs and the supply of energy are included, while the processing, packaging, transport, storage, and recycling phases are excluded. The tool contains information from 2,086 farms with different production systems, 46% of which are certified organic.

Thereby, in the NutriNet-Santé study (4,5), the greenhouse gas (GHG) emissions (CO2-eq) were calculated by multiplying the individual food consumption of each product by the corresponding values of the GHG emissions and conversion factors. The conversion factors allow for the conversion of agricultural products into food products to be able to estimate the environmental impacts associated with food as consumed. Economic allocations by co-product were applied, followed by mass, cooking, and edibility coefficient assignments, enabling the measure of GHG emissions for the ingredient as consumed. Then, by summing all the food consumed, considering the production mode (organic or conventional), the GHG emissions of the overall diet are obtained.

## Supplemental Method 3: Computation of dietary indexes

The nutritional quality of individual diets was assessed using the **PNNS-GS2** (Programme National Nutrition Santé – Guidelines Score 2), which measures adherence to the French dietary recommendations established by the High Council of Public Health in 2017 (6) and launched in 2019. This score includes 13 food groups, classified into two categories:

Healthy foods (to be encouraged), including fruits and vegetables, wholegrain foods, legumes, milk and dairy products, nuts, fish and seafood, and plant-based oils rich in α-linolenic acid; and unhealthy foods (to be limited), including sugary foods, red meat, processed meat, sweetened beverages, alcoholic beverages, and salt.

The reference values are based on the French food-based dietary guidelines established by the High Council of Public Health in 2017 (7). Details regarding the specific food group components and their scoring are provided in **Table 1** below. Reference portion sizes were determined according to typical French consumption patterns (6).

An expert panel established the scoring system, assigning values and thresholds ranging from 0 to 2 points for healthy foods to reflect compliance with recommendations, and from 0 to –2 points for foods to be limited (6). More specifically, high consumption of healthy foods results in positive points, while the absence of consumption may lead to negative points. Conversely, for foods to be limited, high consumption results in negative points, while no consumption does not incur any penalty.

Additionally, the score accounts for organic food consumption for plant-based foods (notably bread and cereals, fruits and vegetables, and legumes) by assigning bonus or penalty points. Weighting was applied to the components according to the strength of evidence for their relationship with health, as determined by an expert panel (6). Furthermore, if total energy intake exceeds estimated energy expenditure by more than 5%, a penalty is applied to the score (PNNS-GS penalized), thus, participants reporting energy intake of more than 105% of their theoretical energy expenditure had their score reduced by the same proportion. As % is a continuous impact of penalty, it may lower the score without a lower limit, which explains the –∞ values. Estimated energy expenditure was based on physical activity level (assessed using the IPAQ questionnaire) and basal metabolic rate using Schofield’s equations (8).

Thus, the final score can theoretically range from –∞ to 14.25.

**Table 1** below provides detailed information on the components, reference servings, and scoring system.

**Table 1.** Programme National Nutrition Santé – guidelines score 2 (PNNS-GS2): components and scoring (9)

| **Dietary components** | **Recommendation** | **Criteria*** | **Score** |
| --- | --- | --- | --- |
| **Fruits and vegetables (weight = 3)** | **At least 5 servings/d, with 1 maximum as juice and 1 maximum as dried** | [0 - 3,5[ | 0 |
|  |  | [3,5 - 5[ | 0,5 |
|  |  | [5 - 7,5[ | 1 |
|  |  | ≥7,5 | 2 |
|  | Prefer organic fruits | Most of the time | 0,5 |
|  |  | Occasionally | 0,25 |
|  |  | Never | 0 |
|  | Prefer organic vegetables | Most of the time | 0,5 |
|  |  | Occasionally | 0,25 |
|  |  | Never | 0 |
| **Nuts** | **A handful/d** | 0 | 0 |
| **(weight =1)** |  | ]0 – 0,5[ | 0,5 |
|  |  | [0,5- 1,5[ | 1 |
|  |  | ≥1,5 | 0 |
| **Legumes** | **At least 2 servings/week** | 0 /sem | 0 |
| **(weight =1)** |  | ]0-2[ /sem | 0,5 |
|  |  | ≥2 /sem | 1 |
|  | Prefer organic legumes | Most of the time | 0,5 |
|  |  | Occasionally | 0,25 |
|  |  | Never | 0 |
| **Whole-grain food** | **Every day** | 0 | 0 |
| **(weight =2)** |  | ]0 - 1[ | 0,5 |
|  |  | [1 - 2[ | 1 |
|  |  | ≥2 | 1,5 |
|  | Prefer organic bread | Most of the time | 0,5 |
|  |  | Occasionally | 0,25 |
|  |  | Never | 0 |
|  | Prefer organic grains | Most of the time | 0,5 |
|  |  | Occasionally | 0,25 |
|  |  | Never | 0 |
| **Milk and dairy products** | **2 servings/d** | [0 - 0,5[ | 0 |
| **(weight =1)** |  | [0,5 - 1,5[ | 0,5 |
|  |  | [1,5 - 2,5[ | 1 |
|  |  | ≥2,5 | 0 |
| **Red meat** | **Limit consumption** | [0 - 500[ g/week | 0 |
| **(weight =2)** |  | [500 - 750[ g/ week | -1 |
|  |  | ≥750 g/ week | -2 |
| **Processed meat** | **Limit consumption** | [0 - 150[ g/ week | 0 |
| **(weight =3)** |  | [150 - 300[ g/ week | -1 |
|  |  | ≥300 g/ week | -2 |
|  | Prefer white ham over other processed meat^1^ | Ratio <50% | 0 |
|  |  | Ratio ≥50% | 0,5 |
| **Fish and seafood** | **2 servings/week** | [0 - 1,5[servings/week | 0 |
| **(weight =2)** |  | [1,5 - 2,5[servings/week | 1 |
|  |  | [2,5 - 3,5[servings/week | 0,5 |
|  |  | ≥3,5 servings/week | 0 |
|  | Fatty fish 1 serving/week | [0 - 0,5[servings/week | 0 |
|  |  | [0,5 - 1,5[servings/week | 1 |
|  |  | ≥1,5 servings/week | 0 |
| **Added fat** | **Avoid overeating** | >16% of EIWA | 0 |
| **(weight =2)** |  | ≤16% of EIWA | 1,5 |
|  | Prefer ALA-rich and olive oil over other oils | Ratio <50% | 0 |
|  |  | Ratio ≥50% | 1 |
|  | Prefer plant fat over animal fat | Ratio >50% | 0 |
|  |  | Ratio ≤50% | 1 |
| **Sugary foods** | **Limit consumption** | <10 % of EIWA | 0 |
| **(weight =3)** |  | [10-15[% of EIWA | -1 |
|  |  | ≥15% of EIWA | -2 |
| **Sweet-tasting beverages**^2^ | **Limit consumption** | 0 mL/d | 0 |
| **(weight =3)** |  | ]0 - 250[ mL/d | -0,5 |
|  |  | [250 - 750[ mL/d | -1 |
|  |  | ≥ 750mL mL/d | -2 |
| **Alcoholic beverages** | **Limit consumption** | >200 g/d | -2 |
| **(weight =3)** |  | ]150-200] g/d | -1.5 |
|  |  | ]100-150] g/d | -1 |
|  |  | ]0-100] g/d | 0 |
|  |  | 0 g/d | 0.5 |
| **Salt** | **Limit consumption** | ≥12 g/d | -2 |
| **(weight =3)** |  | [10-12[ g/d | -1 |
|  |  | [8-10[ g/d | -0.5 |
|  |  | [6-8[ g/d | 0 |
|  |  | <6 g/d | 1 |

EIWA, energy intake without alcohol; ALA, α-linolenic acid. Servings per day unless otherwise stated.

*Servings per day unless otherwise stated.

^1^Conditional: The 0.5 bonus point only occurs if total processed meat consumption is more than 150 g/wk.

^2^Sweetened beverages are specifically sugary, sweetened beverages, artificially sweetened beverages, and fruit juices.

The Comprehensive Diet Quality Index (**cDQI**), as developed by Keaver et al. (10) and applied in the NutriNet-Santé study, provides an overall measure of diet quality by evaluating both plant-based and animal-based dietary components. The cDQI is composed of two sub-scores: the Plant-based Diet Quality Index (**pDQI**) and the Animal-based Diet Quality Index (**aDQI**).

The **pDQI** assesses eleven plant-based food groups, which are classified as either healthy (such as whole grains, fruits excluding juices, vegetables excluding potatoes, nuts, seeds, legumes, plant oils, coffee, and tea) or unhealthy (including refined grains, fruit juices, sugar-sweetened beverages, and sweets or desserts). For each plant-based food group, a score from 0 to 5 is assigned proportionally to the level of consumption relative to a reference intake. For healthy foods, higher consumption up to the recommended value yields higher scores, with a maximum of 5 points. For unhealthy foods, higher consumption results in lower scores, with 0 points assigned if the intake exceeds the recommended limit. The total pDQI is the sum of these eleven components, with a possible range from 0 to 55.

Similarly, the **aDQI** evaluates six animal-based food groups, also classified as healthy (fish and seafood, dairy products, and poultry) or unhealthy (red meat, processed meat, and eggs). The scoring approach mirrors that of the pDQI: higher consumption of healthy animal foods up to the reference value results in higher scores, while higher consumption of unhealthy animal foods leads to lower scores. The total aDQI ranges from 0 to 30.

Reference intakes for each food group are based on international dietary guidelines and scientific evidence (10), with examples including ≥90 g/day for whole grains, ≥150 g/day for fruits and vegetables, ≥100 g/week for fish and seafood, and ≤50 g/week for processed meat. For each component, the score is calculated proportionally to the reference intake using the following formula:

$$Score=\left( \frac{Actual Intake}{Reference Intake} \right)\times5$$

for healthy foods (capped at 5 points), and

$$Score=5-\left( \frac{Actual Intake}{Reference Intake} \right)\times5$$

for unhealthy foods (reduced to 0 if the intake exceeds the recommended limit).

For example, an intake of 90 g of whole grains per day receives 5 points, while 45 g receives 2.5 points; for processed meat, an intake of 0 g per week receives 5 points, while 100 g per week receives 0 points.

The final **cDQI** score is the sum of the **pDQI** and **aDQI**, yielding a total score ranging from 0 to 85, with higher scores indicating better overall diet quality. This detailed scoring system allows for a comprehensive and internationally comparable assessment of both plant and animal diet quality. **Table 2** below provides detailed information on cDQI scoring system.

**Table 2:** Components, Mean Intake, Scoring Standards, and Mean Score for the Comprehensive, Plant-Based, and Animal-Based Diet Quality Index among US Adults Aged 20+ Years, NHANES 1999-2014 (10)

| **Components** | **Mean Intake (SD)*^1^*** | | **Max. Score** | **Standard for Max. Score** | **Standard for Min. Score of 0** | | **Mean Sore (95% CI)** |
| --- | --- | --- | --- | --- | --- | --- | --- |
| **Plant-based Components** | | | | | | | |
| Healthful | | | | | | | |
| Whole grains^2^ | 0.77 (0.55) | | 5 | ≥ 1.5 oz. equiv./1,000 kcal | No whole grains | | 1.27 (1.25, 1.28) |
| Vegetables excluding white potatoes^3^ | 1.16 (0.43) | | 5 | ≥ 1.25 cup equiv./1,000 kcal | No vegetables excluding white potatoes | | 2.30 (2.29, 2.32) |
| Whole fruits^2^ | 0.71 (0.55) | | 5 | ≥ 0.4 cup equiv./1,000 kcal | No whole fruit | | 3.20 (3.16,3.23) |
| Nuts/seeds/legumes^3^ | 0.68 (0.43) | | 5 | ≥ 0.5 oz. equiv./1,000 kcal | No nuts, seeds or legumes | | 3.00 (2.97, 3.02) |
| Vegetable oils^4^ | 17.9 (4.94) | | 5 | Highest quintile | Lowest quintile | | 2.59 (2.56, 2.63) |
| Coffee/tea^4^ | 2.01 (1.74) | | 5 | Highest quintile | Lowest quintile | | 2.58 (2.53, 2.62) |
| Unhealthful | | | | | | | |
| Fruit juices^5^ | 0.29 (0.26) | | 5 | No fruit juices | ≥ 0.35 cup equiv./1,000 kcal | | 3.18 (3.15, 3.20) |
| Refined grains^2^ | 5.63 (1.12) | | 5 | ≤ 1.8 oz. equiv./1,000 kcal | ≥ 4.3 oz. equiv./1,000 kcal | | 3.06 (3.04, 3.08) |
| White potatoes^5^ | 0.34 (0.11) | | 5 | No white potatoes | ⩾0.35 cup equiv./1,000 kcal | | 2.57 (2.56, 2.59) |
| Sugar-sweetened beverages (SSB)^3^ | 1.43 (1.25) | | 5 | No SSB | ≥ 1 cup equiv./day | | 1.34 (1.30, 1.37) |
| Sweets and desserts^4^ | 1.77 (9.75) | | 5 | Highest quintile | Lowest quintile | | 2.49 (2.45, 2.52) |
| Plant-based diet quality index (p**DQI**) | | **55** | | ***range: 0-55*** | | 27.6 (27.4, 27.7) | |
| **Animal-based Components** | | | | | | | |
| Healthful | | | | | | | |
| Fish/seafood^3^ | 0.64 (0.32) | | 5 | ≥ 0.5 oz./1,000 kcal | No fish or shellfish | | 2.94 (2.92, 2.97) |
| Daily^2^ | 1.41 (0.51) | | 5 | ≥ 1.3 cup equiv./1,000 kcal | No dairy | | 2.76 (2.74, 2.78) |
| Poultry^4^ | 1.48 (0.45) | | 5 | Highest quintile | Lowest quintile | | 2.48 (2.44, 2.53) |
| Unhealthy | | | | | | | |
| Processed meats^5^ | 0.85 (0.35) | | 5 | No processed meats | ≥ 1 oz. equiv./1,000 kcal | | 2.87 (2.85, 2.89) |
| Red meats^5^ | 1.66 (0.48) | | 5 | No red meats | ≥ 1.6 oz. equiv./1,000 kcal | | 2.46 (2.44, 2.47) |
| Egg^4^ | 0.60 (0.28) | | 5 | Lowest quintile | Highest quintile | | 2.72 (2.69, 2.75) |
| Animal-based diet quality index **(*a*DQI)** | **30** | | ***range: 0-30*** |  |  | | 16.2 (16.2, 16.3) |
| Comprehensive Diet Quality Index **(*c*DQI)** | **85** | | ***range: 0-85*** |  |  | | 43.8 (43.6, 44.0) |

Abbreviations: NHANES, National Health and Nutrition Examination Survey; SD, Standard Deviation; CI, confident intervals; SSB, sugar-sweetened beverages; pDQI, plant-based Diet Quality Index; aDQI, animal-based Diet Quality Index; cDQI, comprehensive Diet Quality Index.

1.Units are oz. equiv. for mean intake of whole grains, nuts/seeds/legumes, refined grains, fish/seafoods, poultry, processed meats, red meats, and eggs; cup equiv. for mean intake of whole fruits , fruit juices, vegetables excluding white potatoes, white potatoes, and dairy; 8-fluid oz. cup for mean intake of coffee/tea and SSB; and serving for mean intake of sweets and desserts; gram for vegetable oils. The conversion factors for conventional unit to metric unit vary by foods and food groups.(43) The approximate conversion factors are 1 cup equivalent fruits or vegetables is 100 grams; 1 cup equivalent legumes is 175 grams; 1 oz. equivalent whole or refine grains is 30 grams; 1 oz. equivalent fish/seafood, poultry, processed meat, unprocessed red meat, or nuts/seeds is 28.35 grams; 1 cup 8-fluid oz sugar-sweetened beverages, coffee, or tea is 226.8 grams, and 1 serving of sweets and desserts is 30 grams.

2.Scoring is based on scoring standards used in the Healthy Eating Index (HEI)-2015. (23)

3.Scoring is based on scoring standards using in the Alternative Healthy Eating Index (AHEI) adjusted to per 1,000 kcal (24).

4.Scoring is based on the scoring standards used in the Plant-Based Diet Index (PDI) by Satija et al. (19) The quintiles were Q1=13.0, Q2=15.5, Q3=17.6, Q4=19.8, and Q5=22.6 for vegetable oils (grams per 2,000 kcal); Q1=0.38, Q2=0.93, Q3=1.68, Q4=2.38, and Q5=3.43 for tea/coffee (cup equiv. per 2,000 kcal); Q1=6.8, Q2=2.5, Q3=2.0, Q4=1.65, and Q5=1.34 for sweets/desserts (serving per 2,000 kcal); Q1=1.05, Q2=1.22, Q3=1.40, Q4=1.61, and Q5=1.90 for poultry (oz. equiv. per 2,000kcal); and Q1=2.63, Q2=0.85, Q3=0.65, Q4=0.53, and Q5=0.43 for eggs (oz. equiv. per 2,000kcal).

5.Scoring is based on the scoring standards used in the American Heart Association (AHA) diet score based on the AHA 2020 Strategic Goals for Diet,(25) corresponding to 80th percentile of intake among U.S. adults in NHANES 1999-2014.

##

## Supplemental Method 4: Group-based multi-trajectory modeling (GBMTM) method

Group-Based Multi-Trajectory Modeling (GBMTM) is an advanced statistical technique used to identify and analyze distinct groups of individuals who follow similar trajectories across multiple outcomes or behaviors over time (11,12). Unlike conventional Group-Based Trajectory Modeling (GBTM), which models the evolution of a single outcome (for example, meat consumption) in isolation, GBMTM allows for the simultaneous modeling of multiple correlated trajectories within a single analytical framework. GBMTM is particularly useful in understanding the co-development of various related behaviors or conditions. This means that it can identify population subgroups that share similar patterns of change across multiple variables over time.

For example, GBMTM can reveal a group of individuals who simultaneously increase their vegetable intake while decreasing their meat consumption, illustrating a coordinated shift in dietary habits. By jointly modeling these outcomes, GBMTM provides a more holistic and realistic understanding of how different aspects of diet and sustainability evolve together, capturing potential synergies or trade-offs that might be overlooked if each variable were analyzed separately.

The parameters of the GBMTM model are estimated using **Maximum Likelihood Estimation** (MLE). This approach estimates the probability that an individual follows a particular set of trajectories, given their data, and assigns them to the most likely latent class.

GBMTM accommodates missing data through the MLE framework under the assumption that missing values are missing at random (MAR). Participants with incomplete follow-up (e.g., missing dietary data in one or more measurement years) are retained in the analysis if they have at least one non-missing observation across the time points. This approach maximizes the use of available data and reduces potential bias due to attrition. Several criteria are used to determine the optimal number of latent classes, including the Bayesian Information Criterion (BIC), Akaike Information Criterion (AIC), and entropy measures. Then, Individuals are assigned to latent classes based on posterior probabilities, which indicate the likelihood that an individual's observed data corresponds to a particular multi-trajectory pattern.

## Supplemental Method 5: EpiDiet Simulation-Based Nutritional and Epidemiologic Model

EpiDiet ("Evaluate the Potential Impact of a Diet") is a simulation-based model designed to assess the nutritional and epidemiologic risks associated with diet. Like other simulation-based risk assessment models (13–15), it integrates modelling, stochastic methods, and optimization techniques to explore the relationships between diet, nutrition, and the risks of non-communicable diseases. The model's primary goal is to quantify the health risks and benefits arising from dietary changes within a population. EpiDiet is built on the Comparative Risk Assessment framework (16), a widely-used approach by organizations such as the EAT-Lancet Commission (17), the World Health Organization (16), and the Global Burden of Disease Study (GBD) (18) to estimate mortality and disability related to various risk factors, including dietary intake, environmental exposure, and nutrient consumption.

As a structural risk assessment tool, EpiDiet incorporates mechanistic and statistical sub-models, covering a broad range of risk factors including nutrition (food, energy, and nutrient intake, and alcohol consumption), lifestyle (sedentary behaviour, physical activity, smoking), and anthropometrics (age, sex, weight, height). It estimates the impact of dietary changes on morbidity and mortality risks from chronic diseases using the standard Potential Impact Fraction formula for dietary factors. The population is stratified by age and sex to account for variations in diet, disease risk, and relative risks between individuals.

In this study, we applied the EpiDiet model to estimate the impact of dietary pattern trajectories on morbidity and mortality risks for the profiles from 2014 to 2022. The model was parameterized with demographic and mortality data specific to the French population in 2014. Demographic data, including age and sex distributions, were obtained from the National Institute of Statistics and Economic Studies (19). The list of diet-related chronic diseases was derived from the GBD Study 2017 (20) and categorized using the 10th revision of the International Statistical Classification of Diseases and Related Health Problems (ICD-10) (21). Input data included the consumption of fruits, vegetables, whole grains, nuts, seeds, milk, red meats, processed meats, sugar-sweetened beverages, legumes, and energy intake (means and standard deviations). The baseline scenario represented the average dietary intake of each food and beverage group per capita within the whole sample (n=17,187) in 2014. Counterfactual scenarios were constructed based on the dietary patterns of the trajectory profiles.

Disability Adjusted Life Years (DALYs), a measure of disease burden, were estimated as the sum of Years of Life Lost (YLLs) and Years Lived with Disability (YLDs). YLLs were calculated based on the number of premature deaths multiplied by standard life expectancy, with data obtained from the Global Burden of Disease Study (20). YLDs were estimated by multiplying YLLs by an age-, sex-, and disease-specific conversion rate, allowing us to bypass the complex procedure of estimating disease duration through dynamic modeling and conservative assumptions (22,23). The model was parameterized using relative risk (RR) values from the Global Burden of Disease Study 2017 (20) associated with specific dietary patterns to calculate potential reductions in disease burden across the trajectory profiles. By comparing DALYs avoided relative to the baseline scenario across time points, we quantified the potential health gains in each profile.

The analysis was conducted in two steps. First, point estimates of DALYs avoided for each profile were calculated for 2014 and 2022, relative to the reference scenario (whole sample in 2014). Positive DALYs avoided values indicate a lower health risk for the profile compared to the whole sample in 2014, while negative values indicate a higher health risk. Second, the change in DALYs avoided over time (2014–2022) was calculated for each profile in relation to the reference scenario. A positive change indicates that dietary modifications within the profile over time (2014–2022) reduced health risk, whereas a negative change reflects an increase in health risk.

We further examined the contribution of individual food groups to the variation in DALYs avoided over time. For each profile, the model estimated the effect of changes in food group consumption on DALYs avoided, based on dietary exposures relevant to chronic disease risk. These estimations provided insight into the relative influence of each food group on DALY avoided from 2014 to 2022.

## Supplemental Table 1: Sustainability indicators over time (2014-2022) by profile, n = 17,187, NutriNet-Santé Study^1,2^

| **Indicators** | **P0** |  | **P1** |  | **P2** |  | **P3** |  | **P4** |  | **P5** |  |
| --- | --- | --- | --- | --- | --- | --- | --- | --- | --- | --- | --- | --- |
|  | **2014** | **2022** | **2014** | **2022** | **2014** | **2022** | **2014** | **2022** | **2014** | **2022** | **2014** | **2022** |
| **TEI (Kcal/d)** | 2,040  (2,022; 2,058) | 2,005  (1,984; 2,025) | 2,201  (2,158; 2,245) | 2,131  (2,082; 2,181) | 2,218  (2,187; 2,249) | 2,072  (2,035; 2,108) | 2,155  (2,120; 2,190) | 1,996  (1,957; 2,036) | 2,027  (2,003; 2,051) | 2,083  (2,055; 2,111) | 2,307  (2,276; 2,338) | 2,240  (2,207; 2,274) |
| P value | ref | ref | <0.0001 | 0.15 | <0.0001 | <0.0001 | <0.0001 | <0.0001 | 0.36 | <0.0001 | <0.0001 | 0.04 |
| **GHG emissions**  **(kg CO2eq/d)** | 4.33  (4.28;4.37) | 3.81  (3.76;3.86) | 4.85  (4.74;4.96) | 4.30  (4.17;4.43) | 4.39  (4.31;4.47) | 3.94  (3.85;4.04) | 2.19  (2.10;2.28) | 2.09  (1.98;2.19) | 3.56  (3.49;3.62) | 3.24  (3.16;3.31) | 6.53  (6.45;6.61) | 5.63  (5.54;5.72) |
| P value | ref | ref | <0.0001 | 0.69 | 0.17 | 0.17 | <0.0001 | <0.0001 | <0.0001 | <0.0001 | <0.0001 | <0.0001 |
| **PNNS-GS2** | 1.76  (1.69;1.83) | 2.76  (2.68;2.84) | -0.39  (-0.55;-0.22) | 0.19  (0.00;0.39) | 0.71  (0.60;0.83) | 1.71  (1.57;1.85) | 5.43  (5.30;5.56) | 6.60  (6.45;6.76) | 3.98  (3.89;4.08) | 4.46  (4.35;4.57) | -0.40  (-0.52;-0.28) | 0.29  (0.17;0.42) |
| P value | ref | ref | <0.0001 | <0.0001 | <0.0001 | 0.98 | <0.0001 | 0.03 | <0.0001 | <0.0001 | <0.0001 | <0.0001 |
| **DALYs avoided** | -123,450  (-130,479;  -116,421) | -37,087  (-44,193;  -29,981) | -193,525  (-200,569;  -186,481) | -118,125  (-125,003;  -111,246) | -193,725  (-203,583;  -183,866) | -57,333  (-66,672;  -47,994) | 324,272  (312,225;  336,318) | 368,475  (358,363;  378,587) | 236,587  (230,415;  242,760) | 219,867  (213,502;  226,232) | -319,746  (-327,958;  -311,534) | -321,063  (-330,403;  -311,723) |
| **Proportion of organic food** | 0.22  (0.22;0.23) | 0.30  (0.29;0.31) | 0.22  (0.21;0.24) | 0.21  (0.19;0.23) | 0.15  (0.14;0.16) | 0.20  (0.19;0.22) | 0.62  (0.60;0.63) | 0.68  (0.67;0.70) | 0.37  (0.36;0.38) | 0.40  (0.39;0.41) | 0.16  (0.15;0.17) | 0.18  (0.17;0.19) |
| P value | ref | ref | 0.80 | <0.0001 | <0.0001 | 0.0002 | <0.0001 | 0.21 | <0.0001 | <0.0001 | <0.0001 | <0.0001 |
| **cDQI** | 47.77 (47.57;47.97) | 54.15 (53.92;54.39) | 47.72 (47.22;48.22) | 49.64 (49.07;50.20) | 42.82 (42.46;43.18) | 47.07 (46.65;47.49) | 57.17 (56.76;57.57) | 61.32 (60.86;61.78) | 56.62 (56.35;56.90) | 60.50 (60.17;60.82) | 46.16 (45.80;46.51) | 50.41 (50.02;50.79) |
| P value | ref | ref | 0.8645 | <0.0001 | <0.0001 | <0.0001 | <0.0001 | <0.0001 | <0.0001 | <0.0001 | <0.0001 | <0.0001 |
| **aDQI** | 15.04 (14.94;15.14) | 15.86 (15.74;15.97) | 13.49 (13.25;13.74) | 13.49 (13.20;13.77) | 15.56 (15.38;15.73) | 15.50 (15.29;15.71) | 16.21 (16.01;16.41) | 16.04 (15.81;16.27) | 17.27 (17.13;17.41) | 17.71 (17.54;17.87) | 14.11 (13.93;14.28) | 14.71 (14.52;14.90) |
| P value | ref | ref | <0.0001 | <0.0001 | <0.0001 | <0.0001 | <0.0001 | <0.0001 | <0.0001 | 0.0001 | <0.0001 | 0.02 |
| **pDQI** | 32.72 (32.56;32.88) | 38.29 (38.10;38.48) | 34.21 (33.81;34.62) | 36.14 (35.68;36.60) | 27.25 (26.96;27.54) | 31.55 (31.21;31.88) | 40.94 (40.61;41.26) | 45.26 (44.89;45.62) | 39.34 (39.12;39.56) | 42.78 (42.52;43.04) | 32.04 (31.75;32.32) | 35.71 (35.40;36.02) |
| P value | ref | ref | <0.0001 | <0.0001 | <0.0001 | <0.0001 | <0.0001 | <0.0001 | <0.0001 | <0.0001 | 0.0001 | <0.0001 |
| **Proportion of Plant-based Proteins** | 0.30  (0.29;0.30) | 0.33  (0.32;0.33) | 0.29  (0.28;0.29) | 0.29  (0.29;0.30) | 0.25  (0.25;0.26) | 0.30  (0.30;0.31) | 0.58  (0.57;0.58) | 0.61  (0.60;0.62) | 0.35  (0.35;0.36) | 0.37  (0.36;0.37) | 0.23  (0.23;0.24) | 0.25  (0.24;0.25) |
| P value | ref | ref | 0.03 | <0.0001 | <0.0001 | <0.0001 | <0.0001 | 0.71 | <0.0001 | <0.0001 | <0.0001 | <0.0001 |

Abbreviations: GHG, Greenhouse Gas; PNNS-GS2: Programme National Nutrition Santé-Guidelines Score 2 (adherence to French dietary guidelines score); cDQI, Comprehensive Diet Quality Index; pDQI, plant-based Diet Quality Index; aDQI, animal-based Diet Quality Index; TEI, Total Energy Intake; DALYs, Disability Adjusted Life Years.

^1^Values are the least squares means (95%CI) of adjusted scores (sex, age, and total energy intake), except for TEI which was adjusted for sex and age.

^2^P-values are those of β-coefficients of the fixed-effects models. The p-values in 2014 indicate the significance of the difference in baseline score compared to the reference profile (P0). The p-values in 2022 indicate the significance of the difference in score variation over time (2014-2022) compared to the difference in the reference profile (P0).

## Supplemental Table 2: Food group contributions to the variations (2014-2022) of DALYs avoided by profile, n = 17,187, NutriNet-Santé Study^1,2^

| **Food groups** | **P0** |  |  | **P1** |  |  | **P2** |  |  | **P3** |  |  | **P4** |  |  | **P5** |  |  |
| --- | --- | --- | --- | --- | --- | --- | --- | --- | --- | --- | --- | --- | --- | --- | --- | --- | --- | --- |
|  | **2014** | **2022** | **Δ** | **2014** | **2022** | **Δ** | **2014** | **2022** | **Δ** | **2014** | **2022** | **Δ** | **2014** | **2022** | **Δ** | **2014** | **2022** | **Δ** |
| **Fruits** | -44864 | -36451 | **8413** | -58334 | -41334 | **17000** | -56465 | -14402 | **42063** | 30036 | 44505 | **14469** | 94728 | 74349 | **-20379** | -66486 | -76265 | **-9780** |
| **Legumes** | -6701 | 3090 | **9791** | -11506 | -13513 | **-2007** | -14874 | -16091 | **-1217** | 49598 | 40716 | **-8881** | -5557 | 4876 | **10434** | -9333 | -8297 | **1036** |
| **Milk** | -3925 | -8926 | **-5001** | -12646 | -12509 | **137** | 24306 | 29163 | **4856** | -15811 | -13082 | **2729** | -459 | -308 | **1501** | -7192 | -6297 | **895** |
| **Nuts and seeds** | -20935 | -10729 | **10205** | -10627 | -5159 | **5468** | -24222 | -13837 | **10385** | 56478 | 55273 | **-1205** | 5119 | 23011 | **17891** | -26837 | -8425 | **18412** |
| **Processed meat** | 8104 | -4617 | **-12721** | -5411 | -16177 | **-10766** | 4287 | -10361 | **-14648** | 23457 | 14829 | **-8627** | 16330 | -2088 | **-18418** | -17175 | -46889 | **-29714** |
| **Red meat** | 4109 | 32184 | **28074** | -57088 | 4901 | **61989** | 31268 | 87794 | **56526** | 116861 | 150598 | **33737** | 67373 | 75390 | **8018** | -127205 | -105151 | **22054** |
| **SSB** | 6445 | 8164 | **1720** | 8742 | 3837 | **-4906** | -31211 | -23425 | **7786** | 16300 | 17152 | **852** | 6246 | 10411 | **4165** | 1119 | 7066 | **5947** |
| **Vegetables** | -4755 | 3797 | **8552** | -11041 | -11982 | **-941** | -40267 | -41200 | **-934** | 26363 | 22414 | **-3949** | 22495 | 7034 | **-15461** | -13908 | -27698 | **-13791** |
| **Whole-grain products** | -65738 | -26584 | **39153** | -45371 | -31789 | **13583** | -100156 | -60233 | **39922** | 59223 | 71856 | **12633** | 43228 | 40109 | **-3119** | -73830 | -71018 | **2812** |
| **Total DALYs gained** | **-123450** | **-37087** | **86363** | **-193525** | **-118125** | **75400** | **-193725** | **-57333** | **136391** | **324272** | **368475** | **44203** | **236587** | **219867** | **-16720** | **-319746** | **-321063** | **-1317** |

Abbreviations: P0 to P5 refer to the profile 1 to 5, SSB, Sugar sweetened beverages

^1^The deltas represent the difference in DALYs avoided between 2014 and 2022 for each profile, calculated by subtracting the baseline value (2014) from the endpoint value (2022) within each profile.

## Supplemental Figure 1: Selection of the study sample

n=29,195 had valid informed consent in 2022

n=37,685 had completed the organic food questionnaire between June and December 2014

n=37,305 had no missing covariates

n=35,196 were not under-reporter or over-reporter

n=34,453 were not living overseas (for weighting process)

n=29,210 had available data regarding the place of purchase

Final sample in 2014 n=29,210

Among the 29,195 participants: 6,453 completed both 2014 and 2018 FFQs ; 1,639 completed both 2014 and 2022 FFQs ; 9,095 completed all three FFQs (2014, 2018, 2022).

## References

1. Kesse-Guyot E, Castetbon K, Touvier M, Hercberg S, Galan P. Relative validity and reproducibility of a food frequency questionnaire designed for French adults. Ann Nutr Metab. 2010;57(3–4):153–62.

2. Wiley.com [Internet]. [cited 2024 Nov 4]. Student Solutions Manual to accompany Simulation and the Monte Carlo Method, 2nd Edition | Wiley. Available from: https://www.wiley.com/en-cn/Student+Solutions+Manual+to+accompany+Simulation+and+the+Monte+Carlo+Method%2C+2nd+Edition-p-9780470285312

3. Pointereau P, Langevin B, Gimaret M. DIALECTE, a comprehensive and quick tool to assess the agro-environmental performance of farms. Producing and reproducing farming systems New modes of organisation for sustainable food systems of tomorrow 10th European IFSA Symposium, Aarhus, Denmark, 1-4 July 2012 [Internet]. 2012 [cited 2023 Mar 14]; Available from: https://www.cabdirect.org/cabdirect/abstract/20133410218

4. Baudry J, Pointereau P, Seconda L, Vidal R, Taupier-Letage B, Langevin B, et al. Improvement of diet sustainability with increased level of organic food in the diet: findings from the BioNutriNet cohort. The American Journal of Clinical Nutrition. 2019 Apr;109(4):1173–88.

5. Seconda L, Baudry J, Allès B, Boizot-Szantai C, Soler LG, Galan P, et al. Comparing nutritional, economic, and environmental performances of diets according to their levels of greenhouse gas emissions. Climatic Change. 2018 May;148(1–2):155–72.

6. Chaltiel D, Adjibade M, Deschamps V, Touvier M, Hercberg S, Julia C, et al. Programme National Nutrition Santé – guidelines score 2 (PNNS-GS2): development and validation of a diet quality score reflecting the 2017 French dietary guidelines. Br J Nutr. 2019 Aug;122(03):331–42.

7. HCSP. Statement related to the revision of the 2017-2021 French Nutrition and Health Programme’s dietary guidelines for adults [Internet]. Rapport de l’HCSP. Paris: Haut Conseil de la Santé Publique; 2017 Feb [cited 2025 Apr 18]. Available from: https://www.hcsp.fr/explore.cgi/avisrapportsdomaine?clefr=653

8. Schofield WN. Predicting basal metabolic rate, new standards and review of previous work. Hum Nutr Clin Nutr. 1985;39 Suppl 1:5–41.

9. Kesse-Guyot E, Chaltiel D, Fezeu LK, Baudry J, Druesne-Pecollo N, Galan P, et al. Association between adherence to the French dietary guidelines and the risk of type 2 diabetes. Nutrition. 2021 Apr;84:111107.

10. Keaver L, Ruan M, Chen F, Du M, Ding C, Wang J, et al. Plant- and animal-based diet quality and mortality among US adults: a cohort study. Br J Nutr. 2021 Jun 28;125(12):1405–15.

11. Nagin DS, Jones BL, Passos VL, Tremblay RE. Group-based multi-trajectory modeling. Stat Methods Med Res. 2018 Jul;27(7):2015–23.

12. Harvard University Press [Internet]. [cited 2024 Aug 27]. Group-Based Modeling of Development. Available from: https://www.hup.harvard.edu/books/9780674016866

13. Scarborough P, Harrington RA, Mizdrak A, Zhou LM, Doherty A. The Preventable Risk Integrated ModEl and Its Use to Estimate the Health Impact of Public Health Policy Scenarios. Scientifica. 2014;2014(1):748750.

14. Moreira PVL, Baraldi LG, Moubarac JC, Monteiro CA, Newton A, Capewell S, et al. Comparing Different Policy Scenarios to Reduce the Consumption of Ultra-Processed Foods in UK: Impact on Cardiovascular Disease Mortality Using a Modelling Approach. PLoS ONE. 2015 Feb 13;10(2):e0118353.

15. Grieger JA, Johnson BJ, Wycherley TP, Golley RK. Evaluation of Simulation Models that Estimate the Effect of Dietary Strategies on Nutritional Intake: A Systematic Review. J Nutr. 2017 May;147(5):908–31.

16. Murray CJL, Lopez AD. Measuring the Global Burden of Disease. New England Journal of Medicine. 2013 Aug 1;369(5):448–57.

17. Willett W, Rockström J, Loken B, Springmann M, Lang T, Vermeulen S, et al. Food in the Anthropocene: the EAT-Lancet Commission on healthy diets from sustainable food systems. Lancet. 2019 Feb 2;393(10170):447–92.

18. Steinmetz JD, Seeher KM, Schiess N, Nichols E, Cao B, Servili C, et al. Global, regional, and national burden of disorders affecting the nervous system, 1990–2021: a systematic analysis for the Global Burden of Disease Study 2021. The Lancet Neurology. 2024 Apr 1;23(4):344–81.

19. Évolution et structure de la population en 2014 | Insee [Internet]. [cited 2024 Oct 29]. Available from: https://www.insee.fr/fr/statistiques/2862200

20. James SL, Abate D, Abate KH, Abay SM, Abbafati C, Abbasi N, et al. Global, regional, and national incidence, prevalence, and years lived with disability for 354 diseases and injuries for 195 countries and territories, 1990–2017: a systematic analysis for the Global Burden of Disease Study 2017. The Lancet. 2018 Nov 10;392(10159):1789–858.

21. Classification internationale des maladies (CIM) | CépiDc [Internet]. [cited 2024 Oct 29]. Available from: https://www.cepidc.inserm.fr/causes-medicales-de-deces/classification-internationale-des-maladies-cim

22. Barendregt JJ, van Oortmarssen GJ, Vos T, Murray CJ. A generic model for the assessment of disease epidemiology: the computational basis of DisMod II. Population Health Metrics. 2003 Apr 14;1(1):4.

23. Briggs ADM, Cobiac LJ, Wolstenholme J, Scarborough P. PRIMEtime CE: a multistate life table model for estimating the cost-effectiveness of interventions affecting diet and physical activity. BMC Health Services Research. 2019 Jul 16;19(1):485.
